# ATR-FTIR spectroscopy combined with metabolomics to analyze the taste components of *boletus bainiugan* at different drying temperatures

**DOI:** 10.1016/j.fochx.2025.102324

**Published:** 2025-03-04

**Authors:** Guangmei Deng, Honggao Liu, Jieqing Li, Yuanzhong Wang

**Affiliations:** a College of Agronomy and Biotechnology, Yunnan Agricultural University, Kunming 650201, China; b Medicinal Plants Research Institute, Yunnan Academy of Agricultural Sciences, Kunming 650200, China; c Yunnan Key Laboratory of Gastrodia and Fungi Symbiotic Biology, Zhaotong University, Zhaotong 657000, Yunnan, China

**Keywords:** *Boletus bainiugan*, ATR-FTIR spectroscopy, Metabolomics, Taste components prediction

## Abstract

*Boletus bainiugan* has a unique flavor profile, its quality is correlated with metabolites. Herein, ultra-performance liquid chromatography-tandem mass spectrometry (UPLC-MS/MS) is utilized to characterize the free amino acid and organic acid of *Boletus bainiugan* at different drying temperatures. Attenuated total internal reflectance Fourier transform infrared (ATR-FTIR) spectroscopy is employed to identify *Boletus bainiugan* with various treatment and to predicted compounds. The metabolome includes 72 amino acids and 64 organic acids, wherein, 11 important taste components are analyzed the changes with drying temperatures. The residual convolutional neural network (ResNet) model achieves 100 % accuracy for *Boletus bainiugan* with distinct treatment. The partial least squares regression (PLSR) model accurately predicted the contents of 11 compounds with an optimal R^2^_P_ of 0.975 and a best residual predictive deviation (RPD) of 4.404. The ATR-FTIR spectroscopy coupled with metabolomics can be used as a good tool to estimate the taste enhancers of *Boletus bainiugan*.

## Highlights

• Characterizing the metabolomic information of *Boletus bainiugan.*
• Screening and analyzing the taste components at various drying temperatures.
• Successful identification of *Boletus bainiugan* with different drying temperatures.
• Successfully predicted 11 taste components.

## 1 Introduction

Porcini is a fungus in the genus Boletus, family Boletaceae, and is one of the “four king mushrooms”. Due to its unique aroma, taste and flavor characteristics, it has become a favorite wild food in China, Europe and other places (Dong et al., 2021). To satisfy the researchers' curiosity and the demand of consumers, studies have been carried out on the aroma and flavor of many mushrooms. It has been found that the taste flavor is contributed by non-volatile components (metabolite profile), including free amino acids, organic acids, flavor peptides, and other components (Feng et al., 2022). The free amino acids present a highly diverse profile, with valine, tyrosine and tryptophan providing the bitter taste, serine, glutamine giving the sweet taste, as well as glutamic acid and aspartic acid are considered to be the basic components of the umami flavor (Feng et al., 2023). The concentration and composition of organic acids can affect the taste and flavor of mushrooms (Zou et al., 2020). There is still a gap in the research on the flavor of *Boletus bainiugan*, and analyses from different perspectives are important to gain a deeper understanding of the flavor of *Boletus bainiugan*. However, the flavor of *Boletus bainiugan* can be influenced by many factors such as growth conditions, maturity and processing methods (Deng et al., 2023). The drying temperature is one of important factor affecting mushroom quality, which tends to lead to the breakage of chemical bonds, thus leading to the transformation and formation of key compounds (Hou et al., 2022). In actual production, too high a drying temperature may lead to the loss of flavor components, and too low a temperature is likely to lead to mold and deterioration of dried porcini mushroom slices, resulting in poor quality (He, Yang, & Wang, 2023). Thus, the characterization of taste components of *Boletus bainiugan* at different drying temperatures and differentiation between them is imperative.

The metabolomics is considered a useful technique for identifying flavor-related compounds (Chen et al., 2024). The familiar detection methods including liquid chromatography-mass spectrometry (LC-MS), gas chromatography–mass spectrometry (GC–MS), and gas chromatography olfactory (GC-O) detection. Ultra performance liquid chromatography-tandem mass spectrometry (UPLC-MS/MS) based on metabolomics analysis allowed a detailed analysis of the freshness and aroma of *Stropharia granulatus* under different drying methods (Liu et al., 2022). Additionally, spectroscopy, an analytical technique that has the advantage of being efficient, cost-effective and non-destructive. The principle is based on the absorption and vibration of chemical bonds present in organic matter at specific frequencies, which are directly related to the mass of the constituent atoms, the shape of the molecule, and the period of bond hardness (Zhou et al., 2024). Attenuated total internal reflectance Fourier transform infrared (ATR-FTIR) spectroscopy is an effective characterization tool with a fingerprint that reflects the true quality of the sample (Dong et al., 2023). It has a strong potential for applications in content prediction and classification, such as the five metabolites of Katsuobushi (Park et al., 2024), polysaccharides in wine (Baca-Bocanegra et al., 2022), and identification of the origin of *Gastrodia elata* f. glauca (Deng et al., 2025). Studies have illustrated the importance of modeling food flavor and appreciation for the development of quality control, product fingerprinting, counterfeit detection, and traceability studies (Schreurs et al., 2024). The development of a model to recognize drying temperature and predict taste components is one of the necessary research topics in quality control of porcini mushrooms.

**Fig. 1:**
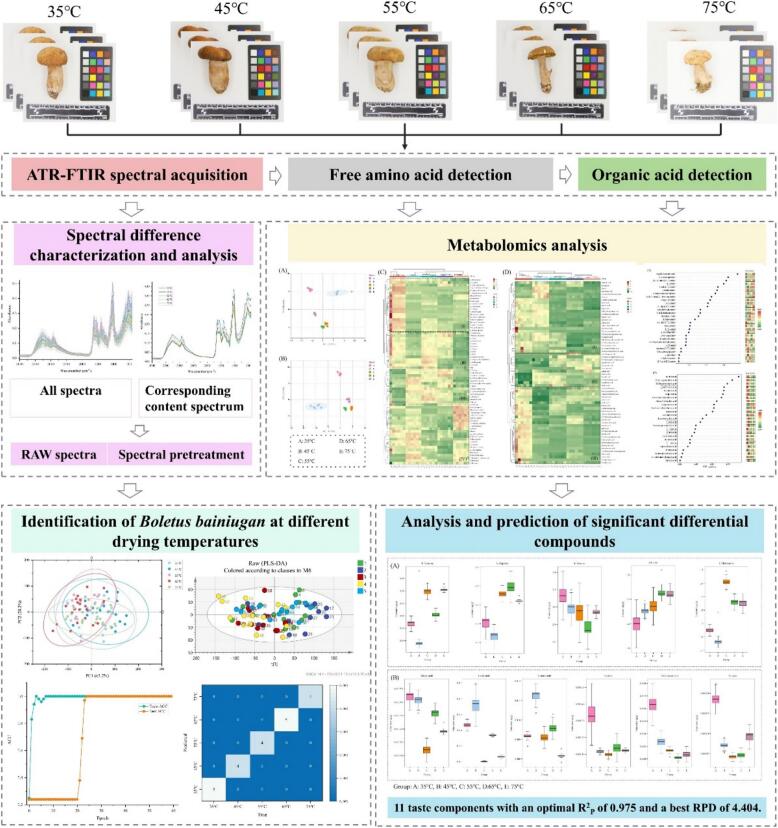
Flowchart of the article.

This present study sought to analyze differences in free amino acids and organic acids of *Boletus bainiugan* at varying drying temperatures by employing metabolomics, in addition to screening the taste components in the differential metabolites, with a view to explaining the impact of drying temperatures on the quality of *Boletus bainiugan*. It is imperative to identify *Boletus bainiugan* with various drying temperatures based on the identified differences in components. Furthermore, the work on the prediction of important taste components is essential for the development of quality evaluation methods for *Boletus bainiugan*. The specific workflow of this study is illustrated in Fig. 1. This study demonstrates the effective application of ATR-FTIR spectroscopy combined with metabolomics for quality assessment of *Boletus bainiugan*.

## 2 Experimental materials and methods

### 2.1 Sample information and drying treatment

The *Boletus bainiugan* were harvested from Yiliang County, Kunming City, Yunnan Province, China, and the sample information is displayed in Table 1. The porcini mushroom fruiting bodies were washed with water after the impurities were removed. Deionized water was used as the final washing solvent. The fruiting bodies were drained of surface moisture and then were dried by a hot-air oven. To investigate the effect of drying temperature on the flavor quality of *Boletus bainiugan*, different drying temperatures were set. The *Boletus bainiugan* were placed in an oven with fixed temperatures (35 °C, 45 °C, 55 °C, 65 °C, 75 °C) and dried to constant weight, the drying time can be manifested in Table 1. The dried samples obtained were pulverized in a pulverizer and sieved through a 100-mesh sieve to produce a fine powder, which was stored in a ziplock bag for spare use.

**Table 1:** The sample information sheet.

Purpose	Drying temperature	Number	Purpose	Drying temperature	Number	Drying time (h)
Discrimination	35 °C	22	Metabolomics analysis, prediction	35 °C	6	67
	45 °C	18		45 °C	6	66
	55 °C	18		55 °C	6	46
	65 °C	21		65 °C	6	22
	75 °C	20		75 °C	6	16
Total		99			30	

### 2.2 Metabolomics detection

#### 2.2.1 Determination of free amino acids and their derivatives

The samples were weighed 50.00 ± 2.50 mg into 2 mL centrifuge tubes and the weighed weight of each sample was recorded. Then 500.00 μL of 70 % methanol aqueous extract (precooled at −20 °C) was added to the weighed samples. The liquid-added samples were vortexed for 3 min and then centrifuged at 12,000 rpm for 10 min at 4 °C. After the above steps, 300.00 *μ*L of the supernatant was aspirated into a 1.5 mL centrifuge tube and allowed to stand for 30 min at −20 °C in a refrigerator. The centrifugation was repeated again for 10 min at the end of the resting time; after that, 200.00 *μ*L of supernatant was pipetted into the injection vial and stored at −20 °C. The data acquisition instrumentation system mainly consisted of UPLC (ExionLCIM AD, https://sciex.com.cn/) and tandem mass spectrometry (MS/MS) (QTRAP® 6500+, https://sciex.com.cn/). The column for UPLC was an ACQUITY BEH Amide column (1.7 μm, 100 mm × 2.1 mm i.d.), the mobile phase for detection was ultrapure water (containing 2 mM ammonium acetate, 0.04 % formic acid) for phase A and acetonitrile for phase B. The column was used for the detection of the analytes. The gradient elution program was 10:90 (*V*/V) for A/B for 0–1.2 min, 40:60 (V/V) for A/B for 1.3–9 min, and 10:90 (V/V) for A/B for 9.1–11 min. The flow rate was 0.40 mL/min, the column temperature was 40 °C, and the injection volume was 2.00 μL. In the mass spectrometry conditions, the electrospray ionization (ESI) temperature was 550 °C, the mass spectrometry voltage was 5500 V in the positive ion mode and − 4500 V in the negative ion mode, and the air curtain gas (CUR) was 35 psi. In the Q-Trap 6500+, each ion pair was scanned and detected based on the optimized declustering potential (DP) and collision energy (CE).

#### 2.2.2 Determination of organic acids and their derivatives

The sample treatment for organic acids was the same as for free amino acids, and the data acquisition system consisted mainly of UPLC and MS/MS. The column used in the present study was an ACQUITY HSS T3 column (1.8 μm, 100 mm × 2.1 mm i.d.), and the detection conditions were as follows: ultrapure water (0.05 % formic acid) was used as the A phase, and acetonitrile (0.05 % formic acid) was employed as the B phase. The elution gradient was 95:5 (*V*/V) for A/B for 0–7.9 min, 5:95 (V/V) for A/B for 8.0–9.5 min, and 95:5 (V/V) for A/B for 9.6–12.0 min. The flow rate was 0.35 mL/min, the column temperature was 40 °C, and the injection volume was 2 μL. Detection was the same as for amino acids in the mass spectrometry assay and the Q-Trap 6500 + .

### 2.3 Spectral acquisition

The ATR-FTIR spectroscopy is mainly equipped with a deuterated tronedium sulfate crystal (DTGS) detector and an ATR accessory with a diamond universal material with a single reflective surface. The instrument was warmed up for two hours prior to spectral collection, then the sample was placed in the collection position and the spectra were acquired by pressing the sample with the ATR diamond. Spectra of *Boletus bainiugan* in the range of 4000–400 cm^−1^ were acquired with a resolution of 4 cm^−1^ by 64 scans in absorbance mode in the OMNIC acquisition software. So as to remove environmentally induced background noise, background spectra need to be collected at half-hourly intervals during the spectral acquisition process. In addition, the assay was performed at a room temperature of 25 ± 2 °C and an air humidity of 40 %.

### 2.4 Data preprocessing

Noise, baseline drift, and light scattering are common problems in spectroscopy that can affect the accuracy of spectral data (He, Yang, & Wang, 2023). Data pretreatment is a fundamental part of chemometrics modeling that removes background noise from spectra to improve model results (Park et al., 2024). In the present study, the spectral data were processed by multiplicative scatter correction (MSC), first derivatives (FD), second derivatives (SD), standard normal variate (SNV), Savitzky-Golay (S-G) and their combinations to process the spectral data.

### 2.5 Chemometrics

Principal component analysis (PCA) is a suitable method for the identification of interrelationships between samples and possible clusters (Pereira et al., 2022). As an unsupervised method, it is widely utilized in studies where spectral differences are used as a basis for classifying samples. In addition to this, the dimensionality of complex datasets can be reduced by extracting essential information based on the spectral attributes of the samples being examined. PCA helps to identify the most important variables in the dataset, which is highly important for building more robust and simpler models (Sánchez-Parra et al., 2024; Yan et al., 2023). In the present study, PCA will be carried out to investigate the clustering of the samples for different temperatures of *Boletus bainiugan*.

PLS-DA is also a common chemometric method that establishes a linear relationship between the independent variable (X) and the dependent variable (Y) to achieve a classification effect (de Andrade et al., 2024). This supervised classification model is a combination of partial least squares regression (PLSR) and classification techniques for recognition, and is capable of handling highly covariant and noisy data (Wang, Chang, et al., 2022). The dataset is divided by the Kennard-Stone (K—S) algorithm into 70 % training set and 30 % testing set to train and validate the model. In the present study, R^2^X, R^2^Y and Q^2^ will be used as indicators for evaluating the model, as well as to check the model fit by 200 permutation tests.

### 2.6 Deep learning

ATR-FTIR spectroscopy provides an overall chemical characterization of the sample, which can lead to suboptimal results from conventional chemometric models due to the overlapping spectral signals of different chemical constituents. To further investigate and highlight the spectral peaks of *Boletus bainiugan*, generalized two-dimensional correlation spectroscopy (2DCOS) was introduced. The 2DCOS image is computed from discrete generalized 2DCOS, where the dynamic spectral intensity at variable *v* is denoted as *Y*, where *t* is the external perturbation and *m* is the spectrum obtained by determining the perturbation *t* at *m* step intervals (Noda, 2014).

(1) $$Y_{\left(v\right)}=\left[\begin{array}{c} \begin{array}{c} Y_{\left(v,t1\right)}\\ Y_{\left(v,t2\right)}\\ \vdots \end{array}\\ \vdots \\ Y_{\left(v,\mathrm{tm}\right)} \end{array}\right]$$

Synchronous 2DCOS has been widely used with superior results in complex mixtures such as food and herbs, such as origin tracing, species identification and food adulteration. In the present study, synchronous 2DCOS images are plotted to build a neural network model, and the synchronous 2DCOS formula is as follows:

(2) $$\Phi_{\left(v1,v2\right)=\frac{1}{m-1}{S_{\left(v1\right)}}^{T}∙S_{\left(v2\right)}}$$

The use of residual convolutional neural network (ResNet) models permits the flow of information through shortcut links to shallow layers, thereby preventing gradient vanishing and exploding. This approach effectively addresses the issue of low model classification performance (Wang et al., 2021). To explore the accuracy of the ResNet model for the recognition of *Boletus bainiugan* with various drying temperatures, this paper draws synchronous 2DCOS image using the original spectra of the samples as the dataset and constructs a ResNet model using this image as the input data. The construction of the ResNet model is shown in Fig. S1. The K—S algorithm is utilized to divide 60 % of the training set, 30 % of the testing set and 10 % of the external validation set before constructing the model. In addition, sensitivity (SEN), specificity (SPE) and efficiency (EFF) are used as evaluation indicators and are calculated as follows:

(3) $$\mathrm{SEN}=\mathrm{TP}/\left(\mathrm{TP}+\mathrm{FN}\right)\;$$

(4) $$\mathrm{SPE}=\mathrm{TN}/\left(\mathrm{TN}+\mathrm{FP}\right)$$

(5) $$\mathrm{EFF}=\sqrt{\mathrm{SEN}\times \mathrm{SPE}}$$

where, TP is true positives, TN is true negatives, FP is false positives, and FN is false negatives (Liu et al., 2023a).

### 2.7 Quantitative model

In this study, a quantitative model of taste components was developed to establish a linear relationship between the ATR-FTIR spectroscopy data (X) and taste compouds content (Y). PLSR is a method of identifying potential variables through the combination of spectral data with sample concentration, thereby accounting for changes in concentration in an efficient manner (Lee et al., 2022). Furthermore, it is also an advanced technique that combines PCA and regression features to effectively solve the major problems encountered when analyzing spectral data, such as covariance and overlapping bands. Based on the advantages of PLSR, it can be employed to explores the linear relationship between spectra and taste component content. Before modeling, the K—S algorithm was used to divide the calibration set (70 %) and prediction set (30 %). The model evaluation standards were mainly the coefficient of determination (R^2^), the root mean square error of prediction (RMSEP), and the residual predictive deviation (RPD). The content of taste components can be effectively predicted using PLSR, which provides a theoretical basis for the subsequent prediction studies of free amino acids and organic acids.

### 2.8 Statistical analysis

The qualitative and quantitative analysis of free amino acids, organic acids and their derivatives were performed by mass spectrometry based on the MWDB database (a database constructed by Metware based on standards). The mass spectrometry data were normalized by weights, and the states 3.5.2 and complexheatmap 2.12.0 function in R version 4.2.0 (www.r-project.org) was used for PCA analysis and cluster heat map analysis of free amino acids and organic acids. Correlation analysis was performed using pearson's correlation test, and variable importance for the projection (VIP) >1, *P* < 0.05 was used as the standard to screen the differential metabolites.

## 3 Results and discussion

### 3.1 ATR-FTIR spectroscopy analysis

**Fig. 2:**
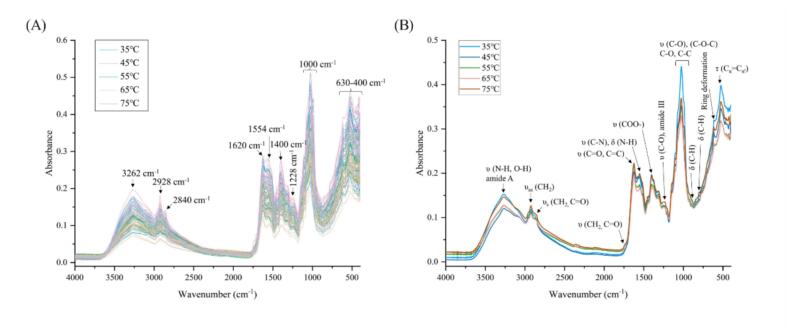
ATR-FTIR Spectroscopy of *Boletus bainiugan*, raw spectra (A), averaged spectra of samples for which free amino acid and organic acid content was determined (B).

The alterations in the chemical composition of *Boletus bainiugan* in response to varying drying temperatures can be elucidated through the examination of its distinctive spectral peaks. The ATR-FTIR spectroscopy of *Boletus bainiugan* at five drying temperatures are illustrated in Fig. 2 A, which demonstrates that the overall spectral peak shapes and the positions of characteristic peaks remain largely consistent. However, a notable distinction is observed in the spectral absorbance intensity. The range of 4000–400 cm^−1^ can be divided into four characterization bands, including 3400–2800 cm^−1^, 1850–1200 cm^−1^, 1200–750 cm^−1^, and 630–400 cm^−1^. The 3400–2800 cm^−1^ broadband originating from O—H stretching vibrations of water, alcohols and phenols, as well as N—H stretching vibrations of the amide A band in proteins and nucleic acid (Bekiaris, Koutrotsios, et al., 2020; Bekiaris, Tagkouli, et al., 2020). This band may also be related to O—H stretching vibrations in triterpenes (Bekiaris, Koutrotsios, et al., 2020). A meticulous examination of the spectra reveals that the asymmetric stretching vibration at 2928 cm^−1^ is ascribed to the aliphatic -CH_2_, while the vicinity of 2840 cm^−1^ is associated with the symmetric stretching vibration of aliphatic -CH_2_ and the C=O stretching vibration (Bekiaris, Koutrotsios, et al., 2020). The characteristic peaks in the 1850–1200 cm^−1^ range are mainly assigned to proteins and amino acids. The C=O stretching vibration of amide I, the C=C and C=O stretching vibration of amino acids, and the N—H bending and aromatic ring deformation of flavonoids are mainly mapped by 1620 cm^−1^ band (Bekiaris, Tagkouli, et al., 2020; Synytsya et al., 2023). In addition, the characteristic peak near 1554 cm^−1^ is related to the amide II of proteins, which is mainly reflected in the N—H bending vibration and C—N stretching vibration. The 1400 cm^−1^ is attributed to the symmetric stretching vibration of COO-, which belongs to the fatty acid and amino acid substances, and it may also reflect the O—H stretching/bending in the C-OH moieties (Bekiaris, Tagkouli, et al., 2020; Li et al., 2020). In this characteristic spectra, 1228 cm^−1^ is unremarkable, whereas it is closely assigned to amide III. This characteristic peak is mainly from the C—O stretching vibration, which may be related to aromatic ethers and phenols (Bekiaris, Koutrotsios, et al., 2020). The subtle characteristic peaks in the strong absorption band near 1000 cm^−1^ are primarily 1147, 1100, 1017 and 918 cm^−1^. These peaks are related to the C-O-C, C—O and C—C stretching vibrations, and the main substances are involved in the *β*-D-glucans in polysaccharides, as well as chitin and *α*-D-glucans (Synytsya et al., 2009). Some minor peaks (e.g., 890 and 760 cm^−1^) are considered to exhibit spectral differences between α- and *β*-glucan (Jozef Sˇandula et al., 1999). In the next feature spectra (630–400 cm^−1^), 620 and 526 cm^−1^ are recognized to be associated with the benzene ring, ring deformation of benzene ring and Cα = Cα’ torsion and ring torsion, respectively (Movasaghi et al., 2008). In conclusion, at various drying temperatures, *Boletus bainiugan* underwent distinct degrees of reaction resulting in differences in the substances in the fruiting bodies, which are reflected in the ATR-FTIR spectroscopy. Although there are significant variations in the spectra of *Boletus bainiugan* from diverse treatments, the spectra overlap severely.

**Fig. 3:**
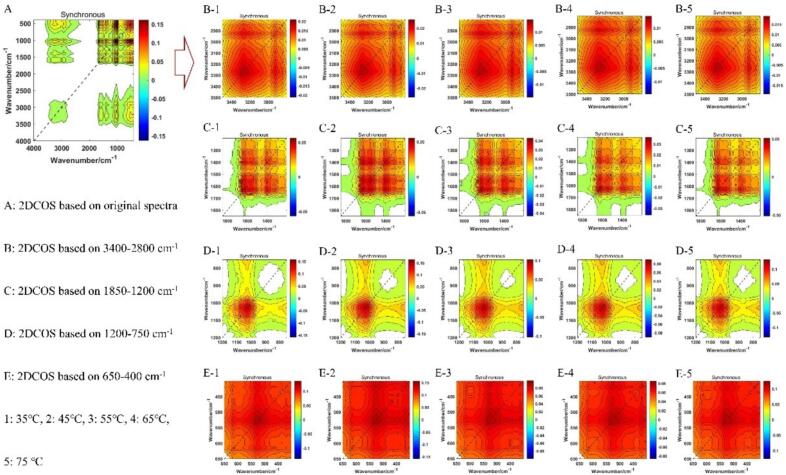
2DCOS images of *Boletus bainiugan* at different drying temperatures.

**Fig. 4:**
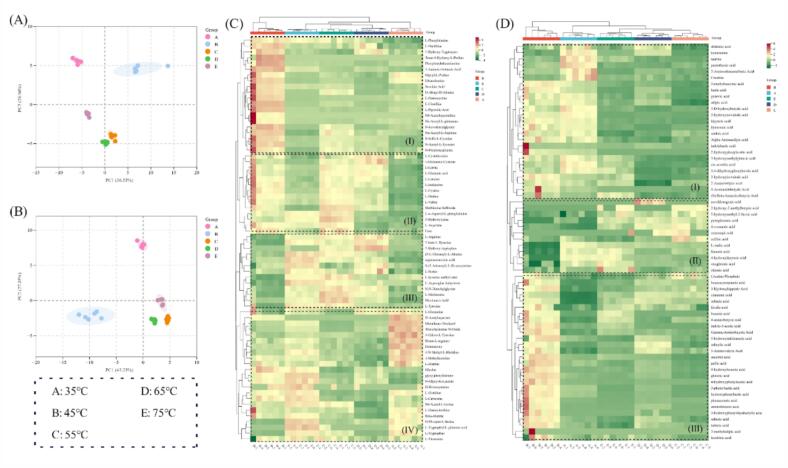
Metabolomics analysis of different drying temperatures. PCA results for free amino acids (A), PCA results for organic acids (B), cluster heatmap analysis for free amino acids (C), and cluster heatmap analysis for organic acids (D).

**Fig. 5:**
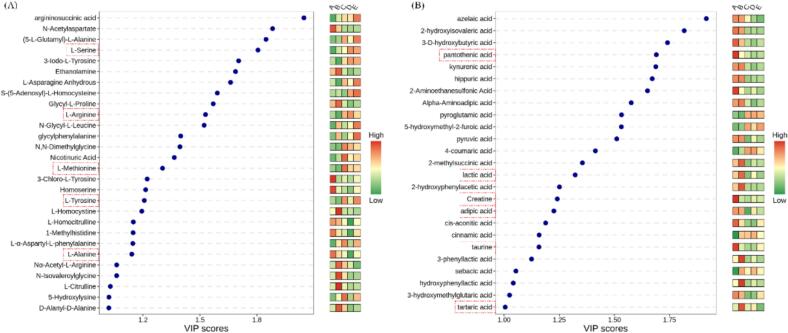
The VIP plot of differential metabolites, free amino acids (A), organic acids (B).

In recent years, 2DCOS has become a prevalent method for characterizing and visualizing discrepancies between different samples. In the present study, 450 synchronous 2DCOS images of the full spectra and feature bands of five drying temperature were computed, as illustrated in Fig. 3. In the 2DCOS images, the auto-peaks represent the peaks on the diagonal line, and the peak area displayed by this peak note the signal intensity of the variable. The cross peak is a symmetrical peak at both sides of the diagonal line. Its signal intensity represents a positive or negative correlation between two variables, namely, the variable of the average spectra and the change in the spectral intensity of the spectral variable for each sample (He, Yang, & Wang, 2023). A comparison of the synchronous 2DCOS images of various feature bands in Fig. 3 reveals that they all characterized the spectral properties and differences of *Boletus bainiugan* at different drying temperatures. Three auto-peaks are identified in Fig. 3 B-1 to B-5, corresponding to 3200 cm^−1^, 2928 cm^−1^ and 2840 cm^−1^, respectively. The discrepancy between the 2928 cm^−1^ and 2840 cm^−1^ peaks are particularly pronounced, indicating that various drying temperatures had distinct effects on the aliphatic compounds in *Boletus bainiugan*. The Fig. 3 C-1 to C-5 is synchronous 2DCOS images that display the feature band of proteins. The variations in the number, positions, and signal intensities of auto-peaks in this range indicate that the synchronous 2DCOS images in this band are capable of characterizing the variations in protein content observed in various samples. Fig. 3 D-1 to D-5 and E-1 to E-5 present the 2DCOS images of the polysaccharides and the phenyl cyclic compounds. Despite the presence of numerous characteristic peaks are cluttered in the raw spectra, the synchronous 2DCOS effectively demonstrates the spectral alterations associated *Boletus bainiugan* with varying drying temperatures. The 2DCOS spectra of the characteristic bands exhibits the variations and differences of the compounds corresponding to each band. Nevertheless, it is still difficult to discriminate different drying temperatures only on the basis of the images, and further pattern recognition methods may be effective in distinguishing between them. Therefore, in the present study, synchronous 2DCOS images with full spectrum (characterizing all information) are employed to establish an image recognition model.

### 3.2 Exploratory analysis

PCA can present a trend in the distribution of data in visual dimensional space, although it cannot be used exclusively as a classification tool (Yan et al., 2023). PCA is used for unsupervised analysis of samples with different drying temperatures, and the results were shown in Fig. S2. In the PCA diagram established by the original spectrum, the PC1 and PC2 accounting for 63.2 % and 28.2 % of the spectral characteristics, respectively. The absence of discernible clustering may be attributed to the presence of a considerable amount of confounding information and noise. Six pretreatment methods are performed on the original spectra to explore whether better spectral quality could improve the PCA results. In the PCA model established after the original and different pretreatment methods, there was no obvious clustering or distinguishable trend in the five drying temperatures. A general pattern can be discerned, whereby temperatures of 35 °C and 45 °C are situated on the positive semiaxis of PC1 in raw and SD-PCA model, while them are distributed on the negative axis of PC1 in other models. The 55 °C and 65 °C samples are mostly distributed in the positive or middle axis of PC2, yet in the negative axis for SD-PCA models. The 75 °C sample distribution is always halfway between PC1 and PC2. Futhermore, in the preprocessed PCA model, PC1 and PC2 have reduced variance interpretation rates for the model. Better spectral quality does not improve the results of PCA. In addition to improving the effect of spectral noise on the PCA model, it is also necessary to consider the variations in substance content that exist between samples. Therefore, further content detection and supervised classification model exploration is necessary.

### 3.3 UPLC-MS/MS analysis

The umami flavor of *Boletus bainiugan* is one of the most important reasons for its popularity among consumers, and it is related to compounds such as free amino acids, organic acids, 5′-nucleotides, etc. (Feng et al., 2022). Spectral analysis reflects the changes of these compounds in *Boletus bainiugan* with different drying temperatures. ATR-FTIR spectroscopy also presents inter- and intra-group variations under diverse treatments. To ascertain whether this phenomenon is associated with the content, a mixed 30 samples were selected for the determination of free amino acid and organic acid content by UPLC-MS/MS targeting. Additionally, the ATR-FTIR spectroscopy data was collected and the chemical bonds or functional groups that are identified in the spectra are found to be consistent with the findings presented in section 3.1 (Fig. 2 B). The averaged spectra demonstrate a differential change in absorbance from 1800 to 1200 cm^−1^, which highlights the significance of this band.

#### 3.3.1 Analysis of free amino acids and derivatives

This study aimed to gain insights into the metabolomic changes of *Boletus bainiugan* at different drying temperatures. The metabolites of *Boletus bainiugan* at various drying temperatures were identified using targeted metabolomics on an UPLC-MS platform. The raw data are converted to the appropriate format and processed using MultiQuant 3.0.3 and Analyst 1.6.3 software, resulting in the generation of the total ion current (TIC) and a multiplex diagram of the multiple reaction monitoring mode (MRM), as illustrated in Fig. S3 A and B. The TIC plot displays a high overlap of the curves for distinct quality control (QC) samples, indicating the stability and reproducibility of the detection results. In the present study, 71 amino acids and their derivative components are identified, and the component types and contents are manifested in Table S1. PCA is a form of unsupervised pattern recognition in multivariate statistical analysis that provides objective sample distributions. PCA is employed to elucidate the distinctions in free amino acid metabolites of *Boletus bainiugan* subjected to disparate drying temperatures. The results in Fig. 4 A suggest that PC1 and PC2 collectively occupies 64.57 % and 31.08 %, respectively, of the original dataset characteristics. There is a clear trend of separation for the five drying temperatures, indicating differences in free amino acid metabolites of *Boletus bainiugan* at various drying temperatures. The 55 °C, 65 °C and 75 °C are distributed in the negative semiaxis of PC2, suggesting that they had similar contents and differed significantly from those between 35 °C and 45 °C. Moreover, 55 °C and 65 °C are more closely distributed, and it is possible that the content of some components changed after high-temperature treatment. A cluster heatmap analysis is employed to ascertain the degree of similarity between distinct drying temperatures. As illustrated in Fig. 4 C, this replicate samples from diverse drying temperatures exhibit a comparable clustering pattern, thereby substantiating the reliability of the data. Initially, the 55 °C, 65 °C, and 75 °C samples are grouped together, subsequently merging with the 35 °C samples and finally with the 45 °C samples. The content of free amino acid differential metabolites manifests distinctions, which could be classified into four categories. In class (I), 45 °C has the most abundant metabolite and also produced richer succinic acid umami components, with less variation in metabolites at the other four temperatures. In class (II), metabolites are richer at 45 °C, 55 °C, and 65 °C, suggesting that temperatures in this range may produce richer bitter amino acids such as L-leucine, L-isoleucine, L-valine, and L-proline. Additionally, these temperatures may contribute to the generation of L-aspartate and L-glutamic acid, which are essential components of the umami profile. In class (III), metabolites are more abundant at 55 °C, 65 °C, and 75 °C, with higher levels of L-serine sweet components and L-methionine sulfur components at these temperatures. In category (IV), relatively higher abundance is found at 35 °C and 75 °C, suggesting that some constituents may have produced derivatives at low and high temperatures. Overall, PCA and cluster analysis reveal significant differences in free amino acid metabolites in different drying temperatures.

**Fig. 6:**
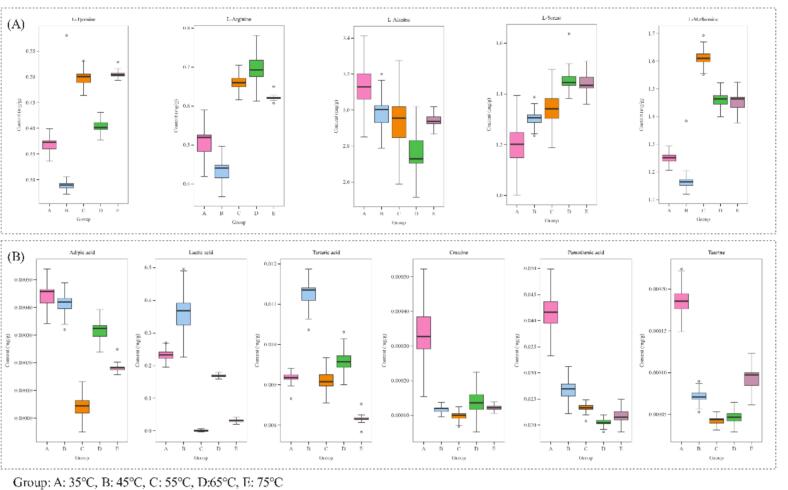
Results of content distribution of important taste components, free amino acids (A), organic acids (B).

In order to gain a deeper understanding of the effect of drying temperatures on free amino acid metabolism, differential metabolites are screened using the criteria of VIP > 1, *P* < 0.05, and a total of 28 differential metabolites are screened. As bespoke in Fig. 5 A, the differential metabolites contained compounds that contributed significantly to the flavor of *Boletus bainiugan*, primarily a sulfur flavor component, two sweet amino acids, and two bitter amino acids. Taste components are essential features that affect the evaluation of food palatability. An in-depth understanding of the changes of important taste components at different drying temperatures is of great significance to the study of the flavor of *Boletus bainiugan*. Changes in the content of five flavor components at distinct drying temperatures are illustrated in Fig. 6 A. L-Arginine and L-methionine have a pattern of decreasing, then increasing, then decreasing. Appropriate temperatures can produce characteristic flavor components, having lower yields at higher temperatures may be due to the loss of amino acids caused by degradation or binding with other macromolecules present in the extracts (Poojary et al., 2017). L-Serine and L-alanine provide sweetness, but the variation with temperature is opposite. The amount of L-serine increases with temperature, suggesting that its production is better favored by high temperature stimulation. This phenomenon is similar to previous findings, which may be attributed to the proteolytic breakdown of proteins and peptides (Yang, Zhang, et al., 2019). The amount of L-alanine decreases with temperature, and it requires mild drying temperatures to perform better. L-Tyrosine provides the bitter flavor that varies from low to high and back again at five temperatures. 55 °C and 75 °C are favorable for its production, and high temperatures can damage the cellular structure of the mushroom, leading to the release of amino acid chemicals from the cells (Yang, Pan, et al., 2019). Nevertheless, the increase in free amino acids at different drying temperatures may be due to varying drying times. In this study, the content variation with temperature is studied only based on five important taste components, yet the specific taste presentation is not yet clear. Uribe et al. (2018) investigated the amino acid content of *Durvillaea antarctica* at diverse drying temperatures. The results indicated that most of the components have a decreasing trend in content during the increase in temperature. In subsequent studies, the introduction of an electronic tongue will help to investigate the changing pattern of porcini taste flavor with temperature even further. Hou et al. (2022) used an electronic tongue to evaluate the flavor parameters of *Suillus granulatus* at different temperatures, and the results bespoke that higher temperatures tended to lead to an increase in bitterness and acidity, which were not conducive to the production of desirable flavors.

#### 3.3.2 Analysis of organic acids and derivatives

The contribution of organic acids to mushroom flavor is well documented, and understanding the metabolism of organic acids in *Boletus bainiugan* can help to promote the progress of research on flavor in *Boletus bainiugan*. There is a significant overlap between the TIC plots and the multi-peak detection plots of the metabolites under MRM (Fig. S3 C and D), and the stability and reliability of the detection method is affirmed. PCA and cluster heatmap analysis are performed according to section 3.3.1 and the results are shown in Fig. 4 B and D. PCA results reveal that PC1 and PC2 explained 43.23 % and 27.03 % of the overall sample variance, respectively. Consistent with the results for amino acid metabolites, different drying temperatures are recognized. The 35 °C and 45 °C have a greater tendency to split, and 55 °C, 65 °C and 75 °C clustered similarly, with 55 °C and 65 °C showing some separation from 75 °C. This distribution is correlated with the concentration of organic acids at distinct drying temperatures, with the concentrations ranked from largest to smallest as 45 °C > 75 °C > 65 °C > 55 °C > 35 °C (Table S2). Clustering heat map analysis is demonstrated that the clustering trend of organic acids is consistent with the results of free amino acids. Organic acids can be categorized into three groups, and in the class I and III, 45 °C has richer concentration variations, which contain high levels of taste components, such as tartaric acid, lactic acid, and adipic acid.

The screening index in section 3.3.1 is used to screen 25 differential metabolites from 64 organic acids components, which contained 6 taste enhancing components, as manifested in Fig. 5 B. The Fig. 6 B displays the changes in the concentration of taste components at various drying temperatures, and the changes can be categorized into two types. Adipic acid, creatine, pantothenic acid and taurine all exhibit a decreasing trend with increasing temperature. Creatine, as a key taste active ingredient, enhances the mouthfeel of porcini flavor. This trend of compositional changes is consistent with the findings of Yang et al. (Yang, Zhang, et al., 2019). Creatine forms creatinine after non-enzymatic conversion by elimination of water and formation of a ring structure under heating conditions, and this conversion promotes the texture of porcini mushrooms (Yang, Pan, et al., 2019). Adipic acid, pantothenic acid and taurine are less analyzed in food flavor studies. It has been suggesting that exposure of porcini mushrooms to high temperatures induces a Maillard reaction that affects flavor compounds. It is also important to consider that prolonged high temperature treatment can severely impair the concentration of other characteristic flavor compounds and key nutrients in porcini mushrooms (Liu et al., 2022). Lactic acid and tartaric acid generally manifest an increasing and then decreasing phenomenon. Lactic acid provides the taste sensation of sourness, but also may be a prerequisite for the formation of the fruity flavor of porcini mushrooms (Wang, Jing, et al., 2022). Tartaric acid has been mainly studied in wine in previous studies, where it contributes to the mouthfeel of wine and may also be a precursor component of the aroma components of wine (Zhao et al., 2023). The present study provides a preliminary explanation of the changes in 11 important taste enhancers in varying drying temperatures. Overall, no individual organic acid or free amino acid has a unique flavor, and the overall taste presentation of *Boletus bainiugan* needs to be evaluated for more taste-contributing components (Yang, Pan, et al., 2019). The introduction of multiple perspectives and data is necessary in subsequent analyses. The study of taste components requires the consideration of taste activity value (TAV) and equivalent umami concentration (EUC) as indicators for assessing the intensity of taste. Feng et al. (2023) employed six methods such as e-tongue, sensory analysis, high performance liquid chromatography (HPLC), and the EUC value method to comprehensively evaluation of taste compounds in 12 edible mushrooms. In conclusion, the present study lays the foundation for the research on the flavor components of *Boletus bainiugan*.

### 3.4 Multimode recognition of ATR-FTIR spectroscopy of *boletus bainiugan* at different drying temperatures

Combined with spectra, free amino acid and organic acid analyses, there are differences in *Boletus bainiugan* between distinct drying temperatures, necessitating discriminates them. PLS-DA can efficiently analyze and process highly correlated variables this approach is more common in spectral data processing (He, Lin, et al., 2023). In the present study, a PLS-DA model is constructed based on the ATR-FTIR spectral data of 99 *Boletus bainiugan* with various drying temperatures, and the effects of different pretreatment methods on the model are also compared. Table 2 presents the outcomes of the PLS-DA models constructed in accordance with the distinct pretreatment methods. All PLS-DA models display interesting results. The modeling results obtained by the MSC, SNV and S-G smoothing methods that focus on eliminating, correcting spectral scattering and noise problems are similar in accuracy to the models constructed from the original spectra. The results suggest that these methods did not serve the preprocessing purpose in the spectral data of *Boletus bainiugan* at distinct drying temperatures. This may be due to the fact that the scattering correction reduces the useful scattering information related to the attribute of interest, which leads to poor modeling results (Mishra et al., 2021). Additionally, the effects of these pretreatments may be species-dependent. In previous studies, the outcome of using MSC, SNV, and S-G when modeling produced suboptimal results (Liu et al., 2023b; Yan et al., 2022). This reminds researchers to use MSC, SNV and S-G preprocessing means with caution when analyzing spectral data related to *Boletus bainiugan*. The models based on derivative processing all improved the identification accuracy of the samples, with the test set ACC can over 84 %. It is worth noting that the results are not effectively improved even with the combination of pretreatment. Mishra et al. (2021) explained that in derivative treatment combined with scatter correction produced worse results than derivative treatment only, similar to the outcomes of the present study. In Fig. S4, the wider distribution of samples in the scatter plot may be attributed to the discrepancy in compound content due to different levels of chemical reactions experienced by *Boletus bainiugan* at various drying temperatures. The outcome in Fig. S4 demonstrates that the scatter plots of the better models (FD, SD, FD + MSC, FD + SNV) exhibit consistent results. There is a clear trend in the separation of *Boletus bainiugan* at 35 °C and 45 °C, with 45 °C consistently distributed on the negative axis and 35 °C on the positive axis in the four models. The majority of the *Boletus bainiugan* at 55 °C, 65 °C, and 75 °C overlapped and could not easily be distinguished. Furthermore, the trend of sample distribution of 45 °C → 55 °C → 65 °C → 75 °C → 35 °C is bespoke from negative to positive on t1 or t2 axes, which is consistent with the PCA results of free amino acids and organic acids. This finding confirms that the absorbance difference of spectra is correlated with the free amino acid, organic acid content. Overall, the results of PLS-DA model constructed by FD, FD + MSC and FD + SNV effectively achieved 100 % training set accuracy and 96.97 % test set accuracy. Based on the definitions of RMSECV, RMSEP and Q^2^, FD + MSC is selected as the best preprocessing method with RMSECV of 0.2843524, RMSEP of 0.2186184 and Q^2^ of 0.553. To validate the performance of the model, a 200-permutation test is performed, and the results is manifested that there is an overfitting phenomenon in the model (Fig. S5), and the model performance is poor.

**Table 2:** Results of PLS-DA model.

Pretreatment methods	R^2^Y	Q^2^	RMSECV	RMSEP	Train ACC	Test ACC
Raw	0.43	0.172	0.3591398	0.3211208	78.79 %	63.64 %
FD	0.909	0.502	0.2793076	0.2367244	100 %	96.97 %
SD	0.88	0.6	0.2832964	0.2599372	100 %	84.85 %
S-G	0.43	0.173	0.3590666	0.3209952	78.79 %	63.64 %
MSC	0.425	0.13	0.3784836	0.313369	78.79 %	60.61 %
SNV	0.421	0.122	0.3803044	0.3129672	78.79 %	63.64 %
FD + MSC	**0.911**	**0.553**	**0.2843524**	**0.2186184**	**100 %**	**96.97 %**
FD + SNV	0.931	0.544	0.2974674	0.21827	100 %	96.97 %

**Fig. 7:**
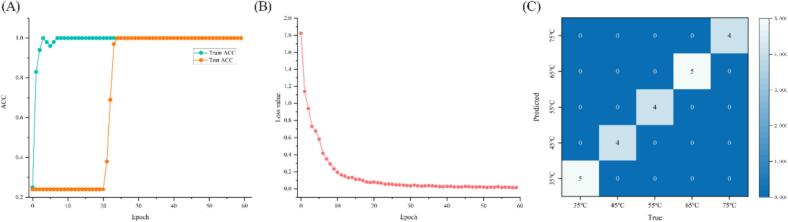
Deep learning results, training and test set results (A), loss function (B), confusion matrix for external validation set (C).

Deep learning is a novel research direction in the field of machine learning, which can presently be applied to the field of chemometrics, after solving some problems in the area of computer vision (Mishra & Passos, 2021). In the present study, a ResNet model is constructed with synchronous 2DCOS images to distinguish *Boletus bainiugan* at different drying temperatures. The results in Fig. 7 A and B demonstrate that the ResNet model can completely and correctly distinguish *Boletus bainiugan* with diverse drying temperatures. When the number of epochs is 24, the training set and test set accuracies stabilize at the 100 % level, indicating that the ResNet model recognition ability is stable. The number of epochs gradually increases and the loss function tends to 0, which represents a good convergence of the model. The model is further verified for classification performance by classifying the external validation set, and the results are emanated in Fig. 7 C. After calculation, the ACC, SEN, and EFF of the ResNet model for the external validation set are all 1. The ResNet model built based on synchronous 2DCOS images does not suffer from overfitting and all samples from the external validation set are classified correctly. This confirms the applicability and stability of the model built from synchronous 2DCOS images as described earlier. The raw data of the ResNet model can support the classification performance of the model, which is shown in sheet 1 in the supplementary material 2. Hence, the synchronous 2DCOS-ResNet model established in this study has superior recognition accuracy and stability. This study explores the ability of chemometrics and deep learning to recognize *Boletus bainiugan* with different drying temperatures, and obtains the expected results, which provide some application potential for the identification of market drying temperatures.

### 3.5 Prediction of taste component content based on differential metabolites

Mushroom flavor has been one of the key research topics of interest, and accurate prediction models for flavor components will greatly help to accelerate progress in flavor research. Furthermore, precise prediction models will facilitate and standardize existing food evaluation methods and have the potential to complement and replace sensory panel evaluation methods (Schreurs et al., 2024). The 1800–1200 cm^−1^ reflects the chemical bonds and functional groups of the free amino acids and organic acids. Thus, the data in this band is used as X and each of the 11 important taste components as Y for investigating the linear relationship between the spectral data and the concentration of components in the samples. Spectral scattering, baseline drift and other problems affect the modeling results and different preprocessing methods are necessary to carry out. Table 3 and Table S3 reveal the results of 11 important taste components under various pretreatments. In general, R^2^ > 0.8 indicates that the model has good predictive ability. The RPD > 1.4 illustrates the model has predictive ability, 2.0 < RPD < 2.5 indicates that the model is very good at quantification, and RPD > 2.5 shows superior model performance (Miao et al., 2023). Overall, the content prediction of taste components of organic acids has better results, with both R^2^_c_ and R^2^_p_ greater than 0.852 and RPD greater than 2.574. However, the prediction models for L-tyrosine, L-alanine, and L-serine have RPDs of only 1.489 to 1.594. This suggests that these components may need to be optimized and more data incorporated in the development of predictive models. The prediction models for L-arginine and L-methionine have superior results R^2^_p_ of 0.909 and 0.945, and RPD of 3.296 and 3.584, respectively. In conclusion, the PLSR model can achieve the prediction of the content of most of the taste components. The prediction of individual components can realize more accurate prediction work, while it has some limitations for the evaluation application in the market. He et al. (2024) accurately predicted multiple quality attributes of the pigments of *Spinacia oleracea* L. using multi-task convolutional neural network (CNN) in conjunction with hyperspectral imaging data, saving a lot of effort and resources. Furthermore, combining electronic tongue data and chemical composition to predict porcini flavor will be one of the breakthroughs in mushroom sensory science (Schreurs et al., 2024). After measuring more than 200 chemical compositions combined with sensory evaluation data and mapping 180,000 consumer review data, the data was substituted to train different predictive models. The results showed that this approach predicts complex food characteristics and drivers of beer flavor and appreciation (Schreurs et al., 2024). The development of a multi-task model and the addition of multi-source data are essential in subsequent studies. In brief, PLSR has effective results in the study of the prediction of important taste components of *Boletus bainiugan*.

**Table 3:** Optimal PLSR modeling results for important taste components.

Compounds	Best pretreatment methods	R^2^_c_	R^2^_p_	RMSEP	RPD
Amino acid					
L-Tyrosine	SD + MSC	0.979	0.832	0.0399765	1.594
L-Arginine	SD + SNV	0.926	0.909	0.0287531	3.296
L-Alanine	SD + MSC	0.531	0.963	0.0706188	1.489
l-Serine	SD + MSC	0.912	0.865	0.0679701	1.553
L-Methionine	SNV	0.984	0.945	0.0447733	3.584

Organic acid					
Adipic acid	MSC	0.988	0.881	0.0000568594	2.574
Lactic acid	MSC	0.933	0.881	0.0519456	2.719
Tartaric acid	SD + SNV	0.899	0.975	0.000308279	2.940
Creatine	MSC	0.852	0.941	0.0000264335	3.237
Pantothenic acid	SD	0.965	0.968	0.0018003	4.098
Taurine	FD	0.932	0.964	0.0000114612	4.404

## 4 Conclusion

In the present study, ATR-FTIR spectroscopy combined with chemometrics, machine learning, and metabolomics are employed for detailed analysis of *Boletus bainiugan* at different drying temperatures. There are differences in the spectra of *Boletus bainiugan* from distinct treatments, which may be related to the content, and it is necessary to differentiate them. The results manifest that the ResNet model based on synchronous 2DCOS has superior ability to recognize *Boletus bainiugan* with various drying temperatures. Drying temperatures are one of the factors affecting the taste flavor of free amino acids and organic acids of *Boletus bainiugan*. Through the determination of targeted metabolome, 71 free amino acid and 64 organic acid compounds are identified, respectively. After PCA and cluster heatmap analysis, the free amino acid and organic acid contents of diverse drying temperatures have a good separation trend, proving the differences in the concentration of the components. In the present study, it is observed that the differential metabolites have 11 important taste components including L-tyrosine, L-arginine, L-alanine, L-serine, L-methionine, adipic acid, lactic acid, tartaric acid, creatine, pantothenic acid and taurine. There are varying concentration changes with increasing temperature. Additionally, 11 taste components are effectively predicted using ATR-FTIR spectroscopy, and their R^2^_p_ are all above 0.832, and the best RPD can reach 4.404. The spectral and content differences of *Boletus bainiugan* at diverse drying temperatures are objectively analyzed by ATR-FTIR spectroscopy coupled with metabolomics analysis, which provided a quality study and rapid market assessment of *Boletus bainiugan*.

## CRediT authorship contribution statement

**Guangmei Deng:** Writing – review & editing, Writing – original draft, Visualization, Software, Data curation, Conceptualization. **Honggao Liu:** Project administration, Funding acquisition, Conceptualization. **Jieqing Li:** Validation, Data curation, Conceptualization. **Yuanzhong Wang:** Resources, Project administration, Methodology.

## Declaration of competing interest

The authors declare that they have no known competing financial interests or personal relationships that could have appeared to influence the work reported in this paper.

## Data Availability

The data that has been used is confidential.

## Appendix A Supplementary data

Supplementary material 1 Image 1

Supplementary material 2 (Model raw data) Image 2

## References

[bb0005] de Andrade J.C., de Oliveira A.T., Amazonas M.G.F.M., Galvan D., Tessaro L., Conte-Junior C.A. (2024). Fingerprinting based on spectral reflectance and chemometrics – An analytical approach aimed at combating the illegal trade of stingray meat in the Amazon. Food Chemistry.

[bb0010] Baca-Bocanegra B., Martínez-Lapuente L., Nogales-Bueno J., Hernández-Hierro J.M., Ferrer-Gallego R. (2022). Feasibility study on the use of ATR-FTIR spectroscopy as a tool for the estimation of wine polysaccharides. Carbohydrate Polymers.

[bb0015] Bekiaris G., Koutrotsios G., Tarantilis P.A., Pappas C.S., Zervakis G.I. (2020). FTIR assessment of compositional changes in lignocellulosic wastes during cultivation of *Cyclocybe cylindracea* mushrooms and use of chemometric models to predict production performance. Journal of Material Cycles and Waste Management.

[bb0020] Bekiaris G., Tagkouli D., Koutrotsios G., Kalogeropoulos N., Zervakis G.I. (2020). Pleurotus mushrooms content in glucans and Ergosterol assessed by ATR-FTIR spectroscopy and multivariate analysis. Foods.

[bb0025] Chen R., Lai X., Wen S., Li Q., Cao J., Lai Z., Sun L. (2024). Analysis of tea quality of large-leaf black tea with different harvesting tenderness based on metabolomics. Food Control.

[bb0030] Deng G., Li J., Liu H., Wang Y. (2023). Volatile compounds and aroma characteristics of mushrooms: A review. Critical Reviews in Food Science and Nutrition.

[bb0035] Deng G., Li J., Liu H., Wang Y. (2025). Rapid determination of geographical authenticity of *Gastrodia elata* f. glauca using Fourier transform infrared spectroscopy and deep learning. Food Control.

[bb0040] Dong J.-E., Li J., Liu H., Zhong Wang Y. (2023). A new effective method for identifying boletes species based on FT-MIR and three dimensional correlation spectroscopy projected image processing. Spectrochimica Acta Part A: Molecular and Biomolecular Spectroscopy.

[bb0045] Dong J.-E., Zhang J., Zuo Z.-T., Wang Y.-Z. (2021). Deep learning for species identification of bolete mushrooms with two-dimensional correlation spectral (2DCOS) images. Spectrochimica Acta Part A: Molecular and Biomolecular Spectroscopy.

[bb0050] Feng T., Cai W., Chen D., Song S., Yao L., Sun M., Dang Y. (2023). Analysis of umami taste and their contributing compounds in edible fungi based on electronic tongue, sensory evaluation, and chemical analysis. Journal of Food Science.

[bb0055] Feng Y., Xin G., Wei Y., Xu H., Sun L., Hou Z., Sun B. (2022). Comparison of the umami taste and aroma of dried *Suillus granulatus* packed using four different packaging methods. Food Chemistry.

[bb0060] He G., Lin Q., Yang S.-B., Wang Y.-Z. (2023). A rapid identification based on FT-NIR spectroscopies and machine learning for drying temperatures of *Amomum tsao-ko*. Journal of Food Composition and Analysis.

[bb0065] He G., Yang S.-B., Wang Y.-Z. (2023). An integrated chemical characterization based on FT-NIR, and GC–MS for the comparative metabolite profiling of 3 species of the genus Amomum. Analytica Chimica Acta.

[bb0070] He M., Jin C., Li C., Cai Z., Peng D., Huang X., Zhang C. (2024). Simultaneous determination of pigments of spinach (*Spinacia oleracea* L.) leaf for quality inspection using hyperspectral imaging and multi-task deep learning regression approaches. Food Chemistry: X.

[bb0075] Hou Z., Wei Y., Sun L., Xia R., Xu H., Li Y., Xin G. (2022). Effects of drying temperature on umami taste and aroma profiles of mushrooms (*Suillus granulatus*). Journal of Food Science.

[bb0080] Jozef Sˇandula G.K., Kacˇura’kova M., Machova E. (1999). Microbial (1→ 3)-β-d-glucans, their preparation, physico-chemical characterization and immunomodulatory activity. Carbohydrate Polymers.

[bb0085] Lee A., Shim J., Kim B., Lee H., Lim J. (2022). Non-destructive prediction of soluble solid contents in Fuji apples using visible near-infrared spectroscopy and various statistical methods. Journal of Food Engineering.

[bb0090] Li X.-P., Li J., Liu H., Wang Y.-Z. (2020). A new analytical method for discrimination of species in *Ganodermataceae* mushrooms. International Journal of Food Properties.

[bb0095] Liu H., Liu H., Li J., Wang Y. (2023). Rapid and accurate authentication of porcini mushroom species using Fourier transform near-infrared spectra combined with machine learning and Chemometrics. ACS Omega.

[bb0100] Liu S., Liu H., Li J., Wang Y. (2023). Solving the identification problems of bolete origins based on multiple data processing: Take *boletus bainiugan* as an example. Journal of Food Composition and Analysis.

[bb0105] Liu Y., Meng F., Tang P., Huang D., Li Q., Lin M. (2022). Widely targeted metabolomics analysis of the changes to key non-volatile taste components in Stropharia rugosoannulata under different drying methods. Frontiers in Nutrition.

[bb0110] Miao X., Miao Y., Liu Y., Tao S., Zheng H., Wang J., Tang Q. (2023). Measurement of nitrogen content in rice plant using near infrared spectroscopy combined with different PLS algorithms. Spectrochimica Acta Part A: Molecular and Biomolecular Spectroscopy.

[bb0115] Mishra P., Passos D. (2021). A synergistic use of chemometrics and deep learning improved the predictive performance of near-infrared spectroscopy models for dry matter prediction in mango fruit. Chemometrics and Intelligent Laboratory Systems.

[bb0120] Mishra P., Rutledge D.N., Roger J.-M., Wali K., Khan H.A. (2021). Chemometric pre-processing can negatively affect the performance of near-infrared spectroscopy models for fruit quality prediction. Talanta.

[bb0125] Movasaghi Z., Rehman S., ur Rehman D.I. (2008). Fourier transform infrared (FTIR) spectroscopy of biological tissues. Applied Spectroscopy Reviews.

[bb0130] Noda I. (2014). Frontiers of two-dimensional correlation spectroscopy. Part 1. New concepts and noteworthy developments. Journal of Molecular Structure.

[bb0135] Park M., Yu J.Y., Ko J.A., Park H.J. (2024). Application of UV-vis-NIR and FTIR spectroscopy coupled with chemometrics for quality prediction of katsuobushi based on the number of smoking treatments. Food Chemistry.

[bb0140] Pereira S.N.G., Lima A.B.S.D., Oliveira T.D.F., Batista A.S., Jesus J.C.D., Ferrão S.P.B., Santos L.S. (2022). Non-destructive detection of soybean oil addition in babassu oil by MIR spectroscopy and chemometrics. Lwt.

[bb0145] Poojary M.M., Orlien V., Passamonti P., Olsen K. (2017). Enzyme-assisted extraction enhancing the umami taste amino acids recovery from several cultivated mushrooms. Food Chemistry.

[bb0150] Sánchez-Parra M., Fernández Pierna J.A., Baeten V., Muñoz-Redondo J.M., Ordóñez-Díaz J.L., Moreno-Rojas J.M. (2024). Rapid screening of tuna samples for food safety issues related to histamine content using fourier-transform mid-infrared (FT-MIR) and chemometrics. Journal of Food Engineering.

[bb0155] Schreurs M., Piampongsant S., Roncoroni M., Cool L., Herrera-Malaver B., Vanderaa C., Verstrepen K.J. (2024). Predicting and improving complex beer flavor through machine learning. Nature Communications.

[bb0160] Synytsya A., Bleha R., Skrynnikova A., Babayeva T., Čopíková J., Kvasnička F., Klouček P. (2023). Mid-infrared spectroscopic study of cultivating medicinal Fungi Ganoderma: Composition, development, and strain variability of Basidiocarps. Journal of Fungi.

[bb0165] Synytsya A., Míčková K., Synytsya A., Jablonský I., Spěváček J., Erban V., Čopíková J. (2009). Glucans from fruit bodies of cultivated mushrooms *Pleurotus ostreatus* and *Pleurotus eryngii*: Structure and potential prebiotic activity. Carbohydrate Polymers.

[bb0170] Uribe E., Vega-Gálvez A., Vargas N., Pasten A., Rodríguez K., Ah-Hen K.S. (2018). Phytochemical components and amino acid profile of brown seaweed *Durvillaea Antarctica* as affected by air drying temperature. Journal of Food Science and Technology.

[bb0175] Wang G., Jing S., Wang X., Zheng F., Li H., Sun B., Li Z. (2022). Evaluation of the perceptual interaction among Ester odorants and nonvolatile organic acids in baijiu by GC-MS, GC-O, odor threshold, and sensory analysis. Journal of Agricultural and Food Chemistry.

[bb0180] Wang L., Li J.-Q., Li T., Liu H.-G., Wang Y.-Z. (2021). Two-dimensional correlation spectroscopy combined with deep learning method and HPLC method to identify the storage duration of porcini. Microchemical Journal.

[bb0185] Wang X.-Z., Chang Y.-Y., Chen Y., Wu H.-L., Wang T., Ding Y.-J., Yu R.-Q. (2022). Geographical origin traceability of medicine food homology species based on an extract-and-shoot inductively coupled plasma mass spectrometry method and chemometrics. Microchemical Journal.

[bb0190] Yan Z., Liu H., Li J., Wang Y. (2023). Qualitative and quantitative analysis of *Lanmaoa asiatica* in different storage years based on FT-NIR combined with chemometrics. Microchemical Journal.

[bb0195] Yan Z., Liu H., Li T., Li J., Wang Y. (2022). Two dimensional correlation spectroscopy combined with ResNet: Efficient method to identify bolete species compared to traditional machine learning. Lwt.

[bb0200] Yang X., Zhang Y., Kong Y., Zhao J., Sun Y., Huang M. (2019). Comparative analysis of taste compounds in shiitake mushrooms processed by hot-air drying and freeze drying. International Journal of Food Properties.

[bb0205] Yang Y., Pan D., Sun Y., Wang Y., Xu F., Cao J. (2019). 1H NMR-based metabolomics profiling and taste of stewed pork-hock in soy sauce. Food Research International.

[bb0210] Zhao Q., Du G., Wang S., Zhao P., Cao X., Cheng C., Wang X. (2023). Investigating the role of tartaric acid in wine astringency. Food Chemistry.

[bb0215] Zhou X., Li L., Zheng J., Wu J., Wen L., Huang M., Zong X. (2024). Quantitative analysis of key components in Qingke beer brewing process by multispectral analysis combined with chemometrics. Food Chemistry.

[bb0220] Zou S., Wu J., Shahid M.Q., He Y., Lin S., Liu Z., Yang X. (2020). Identification of key taste components in loquat using widely targeted metabolomics. Food Chemistry.

