# Supplementary material for: ATR-FTIR spectroscopy combined with metabolomics to analyze the taste components of boletus bainiugan at different drying temperatures
Source: Food Chem X. 2025 Mar 4;26:102324. doi: 10.1016/j.fochx.2025.102324 (PMC11930190; doi:10.1016/j.fochx.2025.102324)
Supplement: Supplementary file 1 — Supplementary material 1 [file mmc1.docx]

ATR-FTIR spectroscopy combined with to analyze the taste components of *Boletus bainiugan* at different drying temperatures

Guangmei Deng ^a, b^, Honggao Liu^c^, Jieqing Li^a*^, and Yuanzhong Wang^b*^

^a^ *College of Agronomy and Biotechnology, Yunnan Agricultural University, Kunming, 650201, China*

*^b^ Medicinal Plants Research Institute, Yunnan Academy of Agricultural Sciences, Kunming 650200, China*

*^c^ Yunnan Key Laboratory of Gastrodia and Fungi Symbiotic Biology, Zhaotong University, Zhaotong 657000, Yunnan China*

^*^Corresponding author: Jieqing Li & Yuanzhong Wang

**Mr: Jieqing Li**, College of Agronomy and Biotechnology, Yunnan Agricultural University, Kunming, 650201, China. E-mail: lijieqing2008@126.com

**Mr: Yuanzhong Wang**, Medicinal Plants Research Institute, Yunnan Academy of Agricultural Sciences, Kunming 650200, China. E-mail: boletus@126.com

Table S1. Free amino acid content of *Boletus bainiugan* at different drying temperatures (mg/g)

| Number | Compounds | 35℃ | 45℃ | 55℃ | 65℃ | 75℃ | Taste Trait |
| --- | --- | --- | --- | --- | --- | --- | --- |
| 1 | Succinic Acid | 9.64475±0.60682 | 17.51486±3.88158 | 11.44848±0.30396 | 8.2943±0.46894 | 11.25368±0.28054 | Umami |
| 2 | L-Glutamic acid | 6.02552±0.20645 | 8.97318±1.40562 | 9.044±0.25982 | 9.10569±0.28694 | 7.10254±0.13496 | Umami |
| 3 | L-Aspartate | 1.02461±0.03972 | 1.70483±0.19872 | 1.66422±0.06994 | 0.93062±0.07699 | 1.48661±0.04204 | Umami |
| 4 | L-Valine | 0.64588±0.02061 | 0.86751±0.09178 | 0.86722±0.02119 | 0.79843±0.0254 | 0.76016±0.01849 | Bitterness |
| 5 | Urea | 0.0068±0.00073 | 0.0098±0.00058 | 0.01018±0.0007 | 0.00637±0.00055 | 0.00744±0.00076 | Bitterness |
| 6 | L-Tyrosine | 0.36758±0.0138 | 0.33658±0.11961 | 0.49885±0.02094 | 0.403±0.01152 | 0.5072±0.01134 | Bitterness |
| 7 | L-Tryptophan | 1.00865±0.08025 | 0.13303±0.03894 | 0.67826±0.06886 | 0.12809±0.01835 | 0.99531±0.02489 | Bitterness |
| 8 | L-Phenylalanine | 0.23318±0.00796 | 0.29248±0.00637 | 0.27365±0.01117 | 0.25891±0.0143 | 0.28202±0.00708 | Bitterness |
| 9 | L-Arginine | 0.50813±0.03954 | 0.43449±0.02172 | 0.66116±0.02202 | 0.6978±0.03039 | 0.62337±0.01416 | Bitterness |
| 10 | L-Histidine | 1.11023±0.05191 | 1.07173±0.04526 | 0.76786±0.02009 | 0.76184±0.04644 | 1.16263±0.03227 | Bitterness |
| 11 | L-Isoleucine | 0.69718±0.02986 | 0.94598±0.09328 | 0.88018±0.02995 | 0.84146±0.0373 | 0.7543±0.0205 | Bitterness |
| 12 | L-Leucine | 0.76044±0.03781 | 1.03128±0.10902 | 0.97339±0.03297 | 0.96487±0.03102 | 0.81347±0.02791 | Bitterness |
| 13 | Creatine | 0.00033±0.00009 | 0.00012±0.00001 | 0.0001±0.00002 | 0.00014±0.00003 | 0.00012±0.00001 | Bitterness |
| 14 | L-Threonine | 2.19419±0.07609 | 2.18595±0.16527 | 2.22957±0.07723 | 2.14106±0.04061 | 2.26432±0.06952 | Sweetness |
| 15 | L-Serine | 1.18989±0.07021 | 1.30621±0.04976 | 1.3529±0.06481 | 1.47294±0.08333 | 1.45128±0.04318 | Sweetness |
| 16 | L-Proline | 0.57234±0.02563 | 0.99095±0.11293 | 0.94587±0.02236 | 0.84021±0.03236 | 0.80162±0.01721 | Sweetness |
| 17 | Glycine | 3.81313±0.05549 | 4.57499±0.20478 | 3.48495±0.06757 | 3.94842±0.14014 | 4.37525±0.09561 | Sweetness |
| 18 | L-Glutamine | 6.42726±0.20422 | 5.75158±0.79826 | 5.56498±0.15833 | 6.43446±0.21313 | 6.18706±0.13218 | Sweetness |
| 19 | L-Alanine | 3.13549±0.10583 | 3.00569±0.11052 | 2.94191±0.11328 | 2.75698±0.09133 | 2.94469±0.04267 | Sweetness |
| 20 | L-Lysine | 0.44985±0.01723 | 0.56598±0.04593 | 0.5665±0.01734 | 0.55688±0.01696 | 0.50154±0.0066 | Sweetness |
| 21 | L-Methionine | 1.25141±0.01877 | 1.19689±0.09234 | 1.61493±0.04639 | 1.46103±0.02081 | 1.45321±0.0247 | Sulphurous |
| 22 | γ-Aminobutyric Acid | 19.33283±0.87304 | 35.83266±1.55019 | 21.79347±0.72667 | 23.57543±0.77654 | 25.61312±0.71062 | Sourness |
| 23 | L-Cystine | 0.01177±0.00143 | 0.19431±0.0478 | 0.17094±0.00682 | 0.09886±0.00511 | 0.06853±0.00233 | Tastelessness |
| 24 | Ethanolamine | 0.053±0.00439 | 0.07236±0.00901 | 0.03106±0.00084 | 0.01787±0.00103 | 0.02456±0.00072 | / |
| 25 | Phosphorylethanolamine | 0.14473±0.0078 | 0.39609±0.06413 | 0.11445±0.00368 | 0.07685±0.00869 | 0.12884±0.00502 | / |
| 26 | (5-L-Glutamyl)-L-Alanine | 0.11544±0.01043 | 0.13028±0.00992 | 0.20398±0.00719 | 0.16342±0.01666 | 0.22273±0.00938 | / |
| 27 | Glutathione Oxidized | 4.72075±0.24182 | 1.49436±0.27697 | 0.59387±0.03297 | 1.42156±0.07905 | 2.81763±0.08367 | / |
| 28 | D-Alanyl-D-Alanine | 0.03042±0.00292 | 0.08662±0.02326 | 0.0559±0.0086 | 0.01254±0.00053 | 0.02198±0.00047 | / |
| 29 | 3-Iodo-L-Tyrosine | 0.00036±0.00002 | 0.00019±0.00005 | 0.00054±0.00003 | 0.00067±0.00007 | 0.00064±0.00004 | / |
| 30 | 3-Chloro-L-Tyrosine | 0.00301±0.0004 | 0.00084±0.0003 | 0.00118±0.0001 | 0.0008±0.00012 | 0.00131±0.00007 | / |
| 31 | glycylphenylalanine | 0.00436±0.00053 | 0.00794±0.00057 | 0.00491±0.0002 | 0.00651±0.0007 | 0.00945±0.00014 | / |
| 32 | Trimethylamine N-Oxide | 0.00058±0.00007 | 0.00014±0.00004 | 0.00006±0.00001 | 0.00019±0.00002 | 0.00028±0.00001 | / |
| 33 | Homoserine | 1.06232±0.04788 | 0.70295±0.01603 | 0.64013±0.04478 | 0.54464±0.05566 | 0.76602±0.03878 | / |
| 34 | L-Cystathionine | 3.31565±0.16957 | 3.53853±0.12061 | 3.47742±0.11777 | 3.60471±0.08103 | 3.21336±0.06428 | / |
| 35 | L-tyrosine methyl ester | 0.0005±0.00003 | 0.00025±0.00003 | 0.00067±0.00005 | 0.00044±0.00005 | 0.00061±0.00002 | / |
| 36 | Nα-Acetyl-L-Arginine | 0.02273±0.00198 | 0.03959±0.00093 | 0.03178±0.00308 | 0.02419±0.00178 | 0.01686±0.00066 | / |
| 37 | Trans-4-Hydroxy-L-Proline | / | 0.00382±0.0019 | / | / | / | / |
| 38 | N8-Acetylspermidine | / | 0.00526±0.00516 | / | / | / | / |
| 39 | 5-Hydroxy-tryptophan | / | / | 0.02311±0.00374 | 0.04706±0.00422 | 0.00215±0.00527 | / |
| 40 | D-Homocysteine | 0.00011±0.00026 | 0.00034±0.00038 | 0.00011±0.00027 | 0.0001±0.00024 | 0.00064±0.00032 | / |
| 41 | L-Homocystine | 0.22577±0.07188 | 0.76591±0.28376 | 0.09172±0.0071 | 0.09805±0.0122 | 0.03317±0.00161 | / |
| 42 | L-Tryptophyl-L-glutamic acid | 0.03267±0.01662 | / | 0.01098±0.01723 | / | 0.06378±0.00449 | / |
| 43 | argininosuccinic acid | 1.00548±0.05434 | 1.54941±0.11321 | 1.88886±0.09343 | 1.83311±0.17213 | 2.32579±0.09836 | / |
| 44 | S-(5-Adenosyl)-L-Homocysteine | 0.42303±0.02271 | 0.4324±0.06523 | 0.40603±0.02588 | 0.5167±0.02609 | 0.54548±0.00922 | / |
| 45 | O-Phospho-L-Serine | 0.09474±0.00751 | 0.04789±0.00222 | 0.06108±0.00308 | 0.06647±0.00586 | 0.10901±0.00276 | / |
| 46 | Nα-Acetyl-L-glutamine | 0.3507±0.03945 | 0.7788±0.39989 | 0.28659±0.01417 | 0.34432±0.02166 | 0.30686±0.00908 | / |
| 47 | Nicotinuric Acid | 0.00063±0.00008 | 0.00159±0.00034 | 0.00441±0.00034 | 0.00259±0.0002 | 0.003±0.00016 | / |
| 48 | N-Isovaleroylglycine | 0.00089±0.00015 | 0.00315±0.00025 | 0.00162±0.00021 | 0.00041±0.00003 | 0.00049±0.00002 | / |
| 49 | N-Acetylaspartate | 0.4049±0.05042 | 0.23381±0.02159 | 0.05277±0.00394 | 0.04325±0.00411 | 0.05325±0.0064 | / |
| 50 | γ-Glutamate-Cysteine | 0.23041±0.03744 | 0.51574±0.19339 | 0.52591±0.0364 | 0.72301±0.08905 | 0.24396±0.01373 | / |
| 51 | N6-Acetyl-L-Lysine | 0.06666±0.00547 | 0.06509±0.00337 | 0.07898±0.00389 | 0.03752±0.00276 | 0.08619±0.0016 | / |
| 52 | S-Sulfo-L-Cysteine | 0.02277±0.00158 | 0.11225±0.02704 | 0.06757±0.00243 | 0.05484±0.00336 | 0.03806±0.00177 | / |
| 53 | N-Glycyl-L-Leucine | 0.02668±0.00201 | 0.0409±0.002 | 0.03225±0.00227 | 0.03696±0.00238 | 0.0454±0.00169 | / |
| 54 | N-Propionylglycine | 0.00755±0.00245 | 0.02092±0.00677 | 0.0142±0.0011 | 0.01021±0.00089 | 0.00464±0.0002 | / |
| 55 | L-α-Aspartyl-L-phenylalanine | 0.00196±0.00011 | 0.00449±0.00029 | 0.00579±0.00024 | 0.00303±0.00019 | 0.00525±0.00018 | / |
| 56 | Glycyl-L-Proline | 0.00267±0.00024 | 0.00323±0.00073 | 0.00197±0.00016 | 0.00151±0.00016 | 0.00176±0.00004 | / |
| 57 | L-Asparagine Anhydrous | 0.86913±0.02811 | 0.76339±0.03207 | 0.9888±0.02594 | 0.91909±0.03098 | 1.05286±0.02664 | / |
| 58 | 5-Hydroxylysine | 0.00161±0.00008 | 0.00192±0.00006 | 0.00235±0.00009 | 0.00172±0.00007 | 0.00214±0.00004 | / |
| 59 | 5-Hydroxy-Tryptamine | 2.79006±0.54381 | 5.05909±0.49732 | 2.04494±0.26055 | 4.43489±0.52763 | 2.77976±0.21051 | / |
| 60 | 3-N-Methyl-L-Histidine | 0.00295±0.00021 | 0.00187±0.00015 | 0.0018±0.00009 | 0.00137±0.00005 | 0.00266±0.00011 | / |
| 61 | 3-Aminoisobutanoic Acid | 3.19803±0.14093 | 5.89773±0.21297 | 3.52608±0.11898 | 3.78886±0.1264 | 4.18538±0.09588 | / |
| 62 | 1-Methylhistidine | 0.01119±0.0004 | 0.00917±0.00045 | 0.00793±0.0003 | 0.00567±0.00039 | 0.00925±0.00019 | / |
| 63 | Beta-Alanine | 0.82715±0.03711 | 0.79183±0.11857 | 0.67716±0.0205 | 0.53256±0.02844 | 0.82999±0.02181 | / |
| 64 | N-Acetyl-L-Tyrosine | 0.02482±0.00147 | 0.10992±0.01605 | 0.06794±0.00402 | 0.05415±0.0031 | 0.04177±0.00132 | / |
| 65 | N, N-Dimethylglycine | 0.00775±0.00132 | 0.00751±0.001 | 0.01212±0.00043 | 0.01116±0.00074 | 0.01046±0.00053 | / |
| 66 | Methionine Sulfoxide | 0.13834±0.00935 | 0.2126±0.02572 | 0.24405±0.00563 | 0.1957±0.00759 | 0.13074±0.00316 | / |
| 67 | Homo-L-arginine | 0.02416±0.00101 | 0.01625±0.00198 | 0.01506±0.00102 | 0.01294±0.00171 | 0.0191±0.00069 | / |
| 68 | L-Pipecolic Acid | 0.02964±0.00328 | 0.08246±0.01331 | 0.02079±0.00243 | 0.02014±0.00234 | 0.02514±0.00024 | / |
| 69 | L-Ornithine | 3.43024±0.0677 | 4.30699±0.07886 | 3.71035±0.11915 | 3.87252±0.39788 | 3.70338±0.05944 | / |
| 70 | L-Citrulline | 0.48152±0.04908 | 2.29924±0.20088 | 0.61131±0.05694 | 0.19668±0.04248 | 0.27578±0.00888 | / |
| 71 | L-Carnosine | 0.01423±0.00092 | 0.01577±0.00072 | 0.01288±0.00035 | 0.01264±0.00052 | 0.01625±0.00026 | / |
| 72 | L-Homocitrulline | 0.0037±0.00016 | 0.00356±0.00082 | 0.0032±0.00018 | 0.0025±0.00013 | 0.00324±0.0001 | / |
|  | Total | 65.3043±1.4136 | 83.7228±7.4266 | 67.2966±1.5004 | 66.6583±2.1409 | 69.9773±1.0711 | / |

Table S2. Organic acid content of *Boletus bainiugan* at different drying temperatures (mg/g)

| Number | Compounds | 35℃ | 45℃ | 55℃ | 65℃ | 75℃ | Taste Trait |
| --- | --- | --- | --- | --- | --- | --- | --- |
| 1 | tartaric acid | 0.00913±0.00024 | 0.0112±0.00046 | 0.00909±0.0002 | 0.00966±0.00036 | 0.00817±0.00022 | Sourness |
| 2 | fumaric acid | 0.05474±0.00483 | 0.00477±0.00545 | 0.0402±0.00385 | 0.02887±0.00107 | 0.05661±0.00731 | Sourness |
| 3 | lactic acid | 0.2343±0.02135 | 0.37275±0.07165 | 0.0011±0.00171 | 0.1688±0.00333 | 0.0309±0.00389 | Sourness |
| 4 | adipic acid | 0.00045±0.00004 | 0.00041±0.00006 | 0.00004±0.00003 | 0.00032±0.00003 | 0.00019±0.00003 | Sourness |
| 5 | benzoic acid | 0.00414±0.00044 | 0.03024±0.00682 | 0.00689±0.00022 | 0.00746±0.00018 | 0.02313±0.00291 | Sourness |
| 6 | gallic acid | 0.00001±0 | 0.00002±0 | / | 0.00001±0 | 0.00001±0 | Bitter |
| 7 | pantothenic acid | 0.04205±0.00309 | 0.02676±0.0018 | 0.02313±0.00128 | 0.02036±0.00091 | 0.02155±0.00144 | Bitter |
| 8 | taurine | 0.00019±0.00002 | 0.00007±0.00001 | 0.00004±0 | 0.00005±0.00001 | 0.00009±0.00001 | Bitter |
| 9 | caffeic acid | 0.00003±0.00001 | 0.00002±0 | 0.00004±0.00001 | 0.00002±0 | 0.00002±0 | Bitter |
| 10 | aminobenzoic acid | 0.00009±0.00001 | 0.00143±0.00059 | 0.00008±0 | 0.00042±0.00001 | 0.00006±0 | Sweetness |
| 11 | salicylic acid | 0.00002±0 | 0.00009±0.00001 | 0.00001±0.00001 | 0.00009±0 | 0.00003±0 | Sweetness |
| 12 | succinic acid | 0.12586±0.01037 | 0.30726±0.02873 | 0.0724±0.00481 | 0.12907±0.00483 | 0.15318±0.01342 | Umami |
| 13 | 2-hydroxyisovaleric acid | 0.00797±0.00059 | 0.00652±0.00057 | 0.00011±0.00001 | 0.00055±0.00002 | 0.00013±0.00001 | / |
| 14 | oleanic acid | 0.00005±0.00001 | 0.00003±0.00001 | 0.00005±0.00001 | 0.00003±0.00001 | 0.00008±0.00003 | / |
| 15 | phenaceturic acid | 0.00011±0.00001 | 0.00059±0.00017 | 0.00011±0.00001 | 0.00014±0.00001 | 0.00007±0 | / |
| 16 | pyruvic acid | 0.00314±0.00029 | 0.00313±0.00063 | 0.00088±0.0001 | 0.00192±0.00023 | 0.00128±0.00019 | / |
| 17 | sebacic acid | 0.00133±0.00008 | 0.00409±0.00023 | 0.00327±0.0001 | 0.00391±0.00016 | 0.00328±0.00033 | / |
| 18 | shikimic acid | 0.00958±0.00229 | 0.00731±0.00157 | 0.00421±0.0005 | 0.00928±0.00096 | 0.00604±0.00106 | / |
| 19 | 5-hydroxymethyl-2-furoic acid | 0.00006±0.00004 | 0.00007±0.00001 | 0.00014±0.00002 | 0.00012±0.00001 | 0.00014±0.00003 | / |
| 20 | oxoglutaric acid | 0.00349±0.00033 | 0.00123±0.00135 | 0.00228±0.00027 | 0.00256±0.00007 | 0.0039±0.00049 | / |
| 21 | benzenepropanoic acid | 0.00003±0.00003 | 0.00104±0.00034 | 0.00054±0.00005 | 0.00066±0.00003 | 0.00032±0.00005 | / |
| 22 | 4-hydroxyhippuric acid | 0.00043±0.00001 | 0.00004±0.0001 | 0.00018±0.00001 | 0.00023±0.00001 | 0.00037±0.00009 | / |
| 23 | 3,4-dihydroxyphenylacetic acid | 0.01261±0.00162 | 0.00793±0.00148 | 0.00229±0.0017 | / | 0.00848±0.00214 | / |
| 24 | pyroglutamic acid | 0.00127±0.0014 | 0.00061±0.00149 | 0.00545±0.00058 | 0.0056±0.00052 | 0.00523±0.00065 | / |
| 25 | 2-hydroxy-2-methylbutyric acid | / | / | 0.00009±0.00001 | / | 0.00006±0.00005 | / |
| 26 | 3-hydroxyphenyl-hydracrylic acid | / | 0.00001±0 | / | / | / | / |
| 27 | crtraconic acid | / | / | 0.00305±0.00336 | 0.00279±0.00306 | 0.00151±0.0037 | / |
| 28 | 3-methyladipic acid | / | 0.00003±0.00007 | / | / | / | / |
| 29 | neochlorogenic acid | / | / | / | 0.00001±0 | / | / |
| 30 | cis-aconitic acid | 0.14758±0.00903 | 0.09788±0.00968 | 0.04867±0.00324 | 0.04227±0.00198 | 0.09389±0.00655 | / |
| 31 | 2-hydroxyphenylacetic acid | 0.00044±0.00004 | 0.0008±0.00051 | 0.00004±0 | 0.00004±0 | 0.00004±0 | / |
| 32 | 3-D-hydroxybutyric acid | 0.00597±0.00047 | 0.00457±0.00065 | 0.00264±0.00007 | 0.00214±0.00011 | 0.00274±0.00023 | / |
| 33 | 3-hydroxyisovaleric acid | 0.03576±0.00115 | 0.04811±0.00382 | 0.02328±0.00121 | 0.02044±0.0007 | 0.03544±0.00467 | / |
| 34 | 3-phenyllactic acid | 0.00732±0.00033 | 0.02106±0.00489 | 0.00014±0.00001 | 0.00502±0.00028 | 0.00035±0.00005 | / |
| 35 | 5-hydroxyindoleacetic acid | 0.00082±0.00015 | 0.00168±0.00064 | 0.00059±0.0001 | 0.00123±0.00007 | 0.00045±0.00007 | / |
| 36 | azelaic acid | 0.00186±0.00015 | 0.00176±0.00037 | 0.00114±0.00015 | 0.00074±0.00006 | 0.00048±0.00007 | / |
| 37 | glutaric acid | 0.00771±0.00112 | 0.02046±0.0007 | 0.00415±0.00049 | 0.00867±0.00038 | 0.00603±0.0005 | / |
| 38 | hippuric acid | 0.00008±0.00001 | 0.00008±0.00001 | 0.00001±0 | 0.00002±0 | 0.00002±0 | / |
| 39 | hydroxyphenyllactic acid | 0.0007±0.00007 | 0.00227±0.0005 | 0.00003±0 | 0.00045±0.00001 | 0.00012±0.00001 | / |
| 40 | indole-3-acetic acid | 0.00089±0.00009 | 0.00277±0.00015 | 0.00078±0.00002 | 0.00082±0.00003 | 0.0016±0.0001 | / |
| 41 | indolelactic acid | 0.00028±0.00004 | 0.00069±0.00102 | 0.00006±0 | 0.00028±0.00002 | 0.00014±0.00001 | / |
| 42 | kynurenic acid | 0.00169±0.00003 | 0.00178±0.00044 | 0.00057±0.00002 | 0.0003±0.00001 | 0.0005±0.00006 | / |
| 43 | kynurenine | 0.00089±0.00012 | 0.00009±0.0001 | 0.0005±0.00003 | 0.00007±0.00001 | 0.00029±0.00002 | / |
| 44 | levulinic acid | 0.03014±0.00132 | 0.03428±0.0053 | 0.02897±0.00062 | 0.03108±0.00056 | 0.03107±0.00157 | / |
| 45 | suberic acid | 0.00047±0.00004 | 0.00099±0.00008 | 0.00035±0.00007 | 0.00067±0.00004 | 0.00032±0.00004 | / |
| 46 | 2-methylsuccinic acid | 0.0096±0.00079 | 0.01189±0.00054 | 0.00556±0.00029 | 0.0071±0.00035 | 0.00657±0.00039 | / |
| 47 | 3-hydroxymethylglutaric acid | 0.0309±0.0025 | 0.02837±0.00323 | 0.01703±0.00056 | 0.018±0.00114 | 0.02568±0.00201 | / |
| 48 | 4-aminobutyric acid | 0.04996±0.0015 | 0.09117±0.01005 | 0.0711±0.00172 | 0.05859±0.00238 | 0.08133±0.00171 | / |
| 49 | 4-coumaric acid | 0.00081±0.00018 | 0.00182±0.0002 | 0.00371±0.00076 | 0.00372±0.00017 | 0.00282±0.00046 | / |
| 50 | 4-hydroxybenzoic acid | 0.00139±0.00007 | 0.00448±0.00057 | 0.00109±0.00013 | 0.00206±0.00009 | 0.00143±0.00016 | / |
| 51 | 4-hydroxyphenylacetic acid | 0.00098±0.00006 | 0.0049±0.00064 | 0.00082±0.00005 | 0.00103±0.00006 | 0.00067±0.00006 | / |
| 52 | L-malic acid | 7.43889±0.23198 | 2.33631±0.35439 | 5.58131±0.16947 | 6.00036±0.20427 | 6.25976±0.23016 | / |
| 53 | adipic acid | 0.00045±0.00004 | 0.00041±0.00006 | 0.00004±0.00003 | 0.00032±0.00003 | 0.00019±0.00003 | / |
| 54 | cinnamic acid | 0.01232±0.00163 | 0.08126±0.00601 | 0.08196±0.0041 | 0.08835±0.00212 | 0.06588±0.00543 | / |
| 55 | (S)-β-Aminoisobutyric Acid | 0.0062±0.00019 | 0.00783±0.00197 | 0.00522±0.00039 | 0.00503±0.00033 | 0.00795±0.00059 | / |
| 56 | Kynurenic Acid | 0.00108±0.00008 | 0.00148±0.00004 | 0.00042±0.00002 | 0.00092±0.00003 | 0.00042±0.00001 | / |
| 57 | 2-Aminobutyric acid | 0.23885±0.01415 | 0.26187±0.01661 | 0.22448±0.0034 | 0.16134±0.00621 | 0.24493±0.00434 | / |
| 58 | 2-Aminoethanesulfonic Acid | 0.01434±0.00242 | 0.00783±0.00035 | 0.00496±0.00046 | 0.00685±0.0002 | 0.00459±0.0002 | / |
| 59 | α-Aminoadipic acid | 2.79645±0.2051 | 2.93032±0.30368 | 2.13±0.08592 | 1.79507±0.13815 | 2.00772±0.15683 | / |
| 60 | kynurenine | 0.00772±0.00081 | 0.00138±0.00047 | 0.00079±0.00013 | 0.00528±0.00011 | 0.00245±0.00007 | / |
| 61 | 4-Acetamidobutyric Acid | 0.20296±0.02249 | 0.42978±0.27004 | 0.05742±0.00298 | 0.08784±0.00613 | 0.10066±0.00257 | / |
| 62 | 5-Aminovaleric Acid | 0.01612±0.00129 | 0.02904±0.00266 | 0.02162±0.00148 | 0.02708±0.00087 | 0.01967±0.00033 | / |
| 63 | 3-Hydroxyhippuric Acid | 0.00001±0.00002 | 0.00022±0.00003 | 0.00013±0.00003 | 0.00011±0.00001 | 0.00006±0.00001 | / |
| 64 | Creatine Phosphate | 0.1869±0.04631 | 0.428±0.07697 | 0.44301±0.09305 | 0.2989±0.05073 | 0.18953±0.02302 | / |
|  | Total | 31.1023±0.8437 | 43.5176±2.2709 | 30.7319±0.9517 | 32.6504±0.6420 | 35.1338±1.0243 | / |

Table S3. PLSR model results with different preprocessing

| Compounds | Pretreatment methods | R2 c | R2 p | RMSEP | RPD |
| --- | --- | --- | --- | --- | --- |
| Amino acid | | | | | |
| L-Tyrosine | Raw | 0.257 | 0.776 | 0.0460228 | 0.933 |
|  | FD | 0.909 | 0.878 | 0.0413607 | 1.436 |
|  | SD | 0.960 | 0.732 | 0.0455743 | 1.537 |
|  | SNV | 0.270 | 0.542 | 0.0573223 | 0.732 |
|  | MSC | 0.268 | 0.542 | 0.0573635 | 0.726 |
|  | SG | 0.256 | 0.774 | 0.0462152 | 0.926 |
|  | **SD+MSC** | **0.979** | **0.832** | **0.0399765** | **1.594** |
|  | SD+SNV | 0.979 | 0.833 | 0.040504 | 1.558 |
| L-Arginine | Raw | 0.823 | 0.767 | 0.0549721 | 1.753 |
|  | FD | 0.908 | 0.889 | 0.0311303 | 2.873 |
|  | SD | 0.830 | 0.759 | 0.0463257 | 1.878 |
|  | SNV | 0.940 | 0.832 | 0.0410813 | 2.423 |
|  | MSC | 0.941 | 0.833 | 0.0409165 | 2.431 |
|  | SG | 0.823 | 0.766 | 0.0549897 | 1.753 |
|  | SD+MSC | 0.926 | 0.908 | 0.02877 | 3.272 |
|  | **SD+SNV** | **0.926** | **0.909** | **0.0287531** | **3.296** |
| L-Alanine | Raw | 0.337 | 0.591 | 0.109132 | 0.896 |
|  | FD | 0.397 | 0.884 | 0.0800636 | 1.214 |
|  | SD | 0.365 | 0.791 | 0.0891358 | 1.082 |
|  | SNV | 0.445 | 0.811 | 0.0843818 | 1.195 |
|  | MSC | 0.597 | 0.863 | 0.075506 | 1.385 |
|  | SG | 0.303 | 0.441 | 0.121301 | 0.736 |
|  | **SD+MSC** | **0.531** | **0.963** | **0.0706188** | **1.489** |
|  | SD+SNV | 0.528 | 0.962 | 0.070649 | 1.480 |
| L-Serine | Raw | 0.743 | 0.570 | 0.0884991 | 1.114 |
|  | FD | 0.758 | 0.721 | 0.0815675 | 1.202 |
|  | SD | 0.901 | 0.825 | 0.0760256 | 1.353 |
|  | SNV | 0.644 | 0.779 | 0.0750837 | 1.246 |
|  | MSC | 0.641 | 0.776 | 0.0754856 | 1.234 |
|  | SG | 0.742 | 0.570 | 0.0885636 | 0.089 |
|  | **SD+MSC** | **0.912** | **0.865** | **0.0679701** | **1.553** |
|  | SD+SNV | 0.913 | 0.863 | 0.0691506 | 1.522 |
| L-Methionine | Raw | 0.960 | 0.899 | 0.0531896 | 2.866 |
|  | FD | 0.945 | 0.900 | 0.060079 | 2.253 |
|  | SD | 0.884 | 0.692 | 0.0945913 | 1.476 |
|  | **SNV** | **0.984** | **0.945** | **0.0447733** | **3.584** |
|  | MSC | 0.984 | 0.942 | 0.045457 | 3.532 |
|  | SG | 0.961 | 0.898 | 0.052947 | 2.891 |
|  | SD+MSC | 0.982 | 0.942 | 0.0509172 | 2.787 |
|  | SD+SNV | 0.981 | 0.943 | 0.0507886 | 2.795 |
| Organic acid | | | | | |
| Adipic acid | Raw | 0.396 | 0.608 | 0.0000988033 | 1.082 |
|  | FD | 0.887 | 0.820 | 0.0000614712 | 2.050 |
|  | SD | 0.958 | 0.742 | 0.0000744293 | 1.734 |
|  | SNV | 0.987 | 0.873 | 0.0000592434 | 2.433 |
|  | **MSC** | **0.988** | **0.881** | **0.0000568594** | **2.574** |
|  | SG | 0.398 | 0.611 | 0.0000984555 | 1.087 |
|  | SD+MSC | 0.890 | 0.828 | 0.0000632463 | 2.108 |
|  | SD+SNV | 0.894 | 0.835 | 0.0000619216 | 2.168 |
| Lactic acid | Raw | 0.510 | 0.830 | 0.0643898 | 1.732 |
|  | FD | 0.904 | 0.841 | 0.0583292 | 1.922 |
|  | SD | 0.896 | 0.599 | 0.0861772 | 1.263 |
|  | SNV | 0.922 | 0.846 | 0.0576128 | 2.406 |
|  | **MSC** | **0.933** | **0.881** | **0.0519456** | **2.719** |
|  | SG | 0.511 | 0.830 | 0.0643475 | 1.733 |
|  | SD+MSC | 0.949 | 0.897 | 0.0500811 | 2.561 |
|  | SD+SNV | 0.949 | 0.900 | 0.0495039 | 2.560 |
| Tartaric acid | Raw | 0.265 | 0.831 | 0.000553708 | 1.004 |
|  | FD | 0.864 | 0.913 | 0.000412996 | 2.100 |
|  | SD | 0.892 | 0.911 | 0.000397829 | 2.137 |
|  | SNV | 0.825 | 0.889 | 0.00038874 | 2.502 |
|  | MSC | 0.821 | 0.876 | 0.000403969 | 2.415 |
|  | SG | 0.263 | 0.831 | 0.000556022 | 0.993 |
|  | SD+MSC | 0.900 | 0.974 | 0.000308861 | 2.932 |
|  | **SD+SNV** | **0.899** | **0.975** | **0.000308279** | **2.940** |
| Creatine | Raw | 0.258 | 0.256 | 0.00007841760 | 0.645 |
|  | FD | 0.817 | 0.900 | 0.00002835460 | 2.816 |
|  | SD | 0.812 | 0.877 | 0.00003196180 | 2.498 |
|  | SNV | 0.851 | 0.941 | 0.00002668770 | 3.210 |
|  | **MSC** | **0.852** | **0.941** | **0.00002643350** | **3.237** |
|  | SG | 0.262 | 0.261 | 0.00007816310 | 0.651 |
|  | SD+MSC | 0.825 | 0.911 | 0.00002703170 | 3.026 |
|  | SD+SNV | 0.827 | 0.912 | 0.00002689460 | 3.053 |
| Pantothenic acid | Raw | 0.596 | 0.407 | 0.00659584 | 0.979 |
|  | FD | 0.915 | 0.983 | 0.00177796 | 4.042 |
|  | **SD** | **0.965** | **0.968** | **0.0018003** | **4.098** |
|  | SNV | 0.870 | 0.926 | 0.0029466 | 2.288 |
|  | MSC | 0.868 | 0.9272 | 0.00293201 | 2.291 |
|  | SG | 0.598 | 0.412 | 0.00655745 | 0.985 |
|  | SD+MSC | 0.929 | 0.983 | 0.00196144 | 3.608 |
|  | SD+SNV | 0.930 | 0.983 | 0.00195391 | 3.632 |
| Taurine | Raw | 0.278 | 0.581 | 0.00003884820 | 0.657 |
|  | **FD** | **0.932** | **0.964** | **0.00001146120** | **4.404** |
|  | SD | 0.937 | 0.940 | 0.00001461720 | 3.391 |
|  | SNV | 0.934 | 0.866 | 0.00001983300 | 2.619 |
|  | MSC | 0.935 | 0.867 | 0.00001969320 | 2.625 |
|  | SG | 0.275 | 0.587 | 0.00003873450 | 0.655 |
|  | SD+MSC | 0.892 | 0.978 | 0.00001235720 | 3.795 |
|  | SD+SNV | 0.890 | 0.977 | 0.00001246620 | 3.759 |


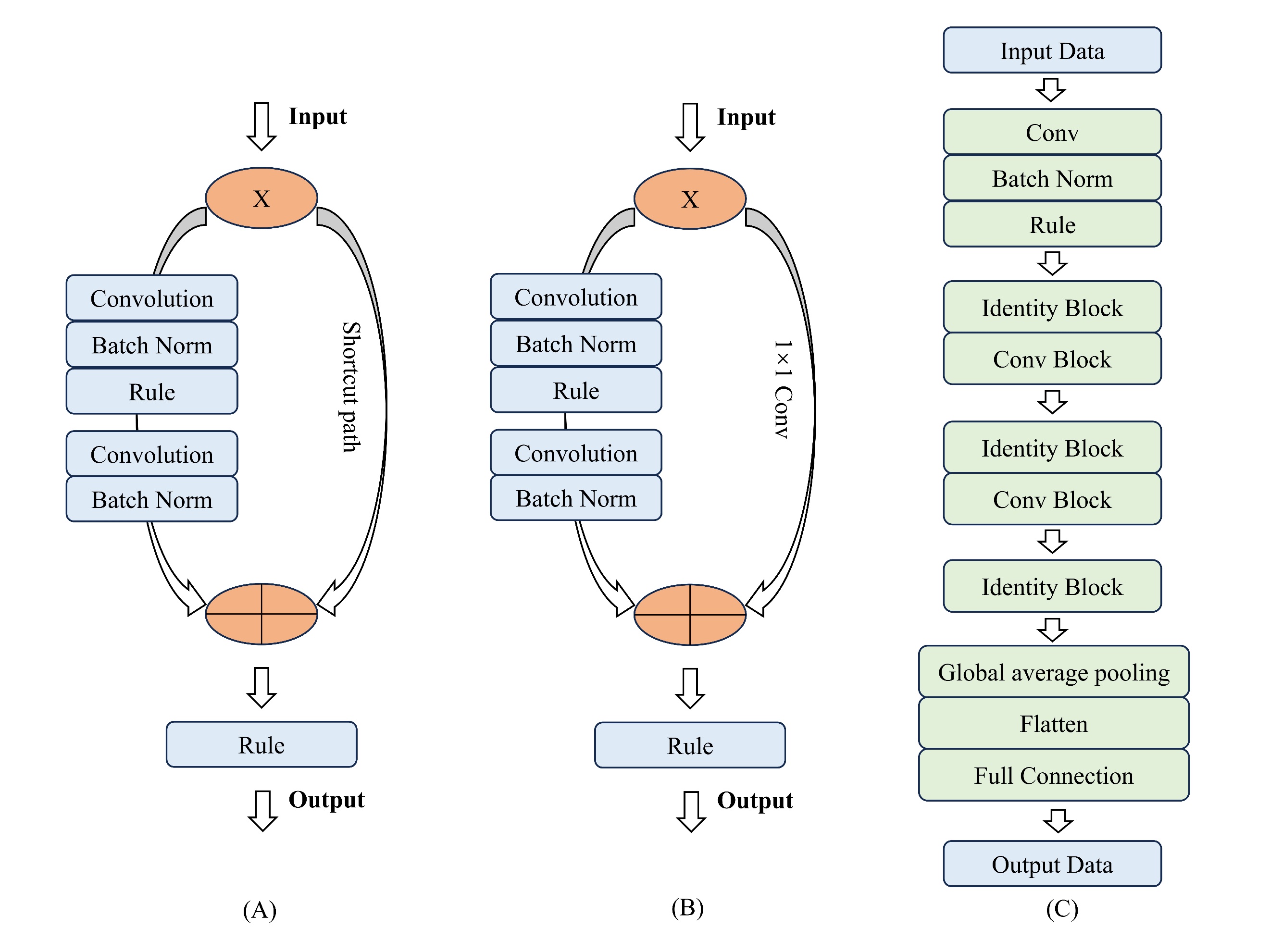


Fig. S1. Residual convolutional neural network model, the structure of identity block (A), the structure of convolutional block (B), the structure of ResNet model (C).


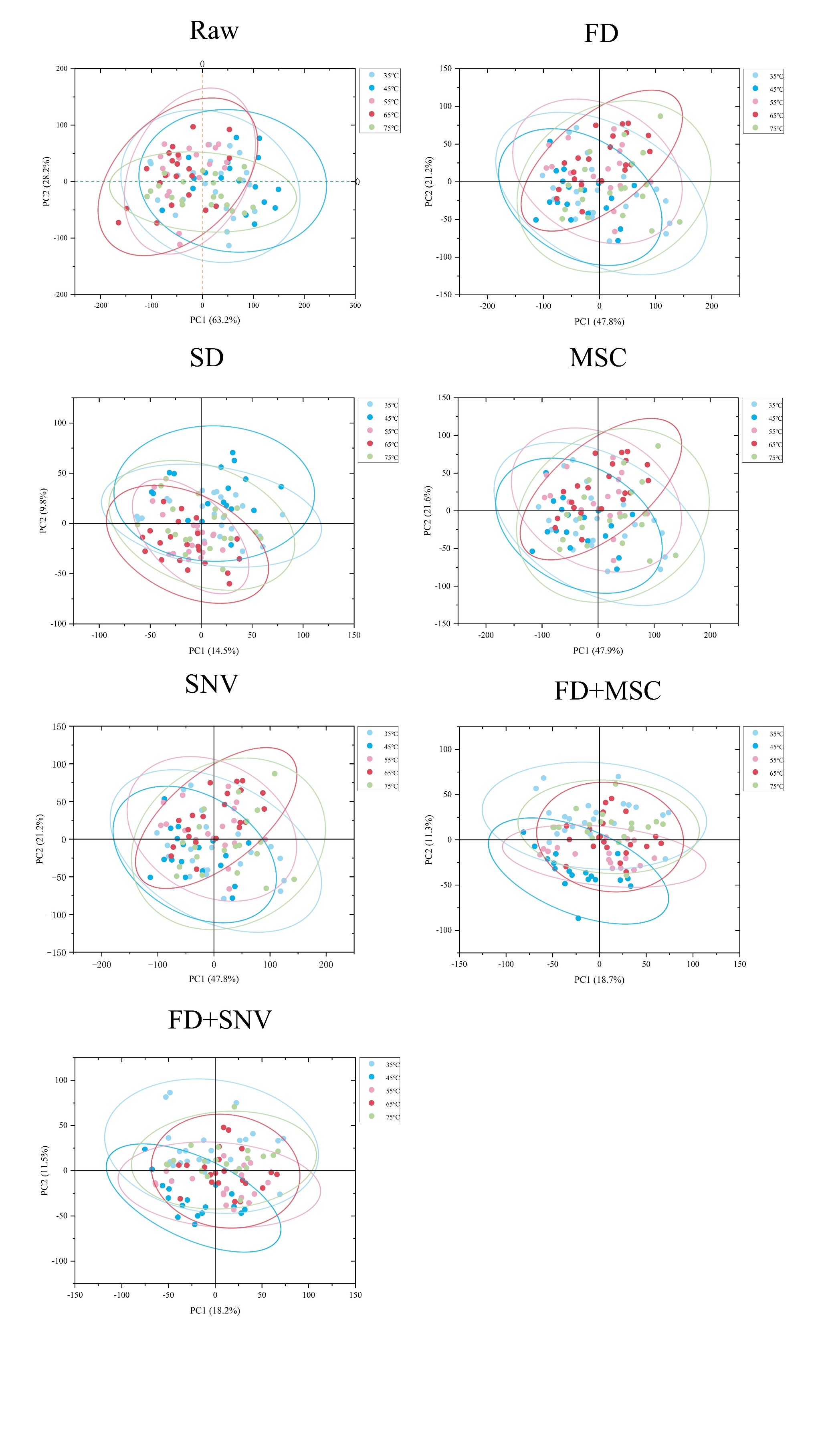


Fig. S2. PCA results with different preprocessing.


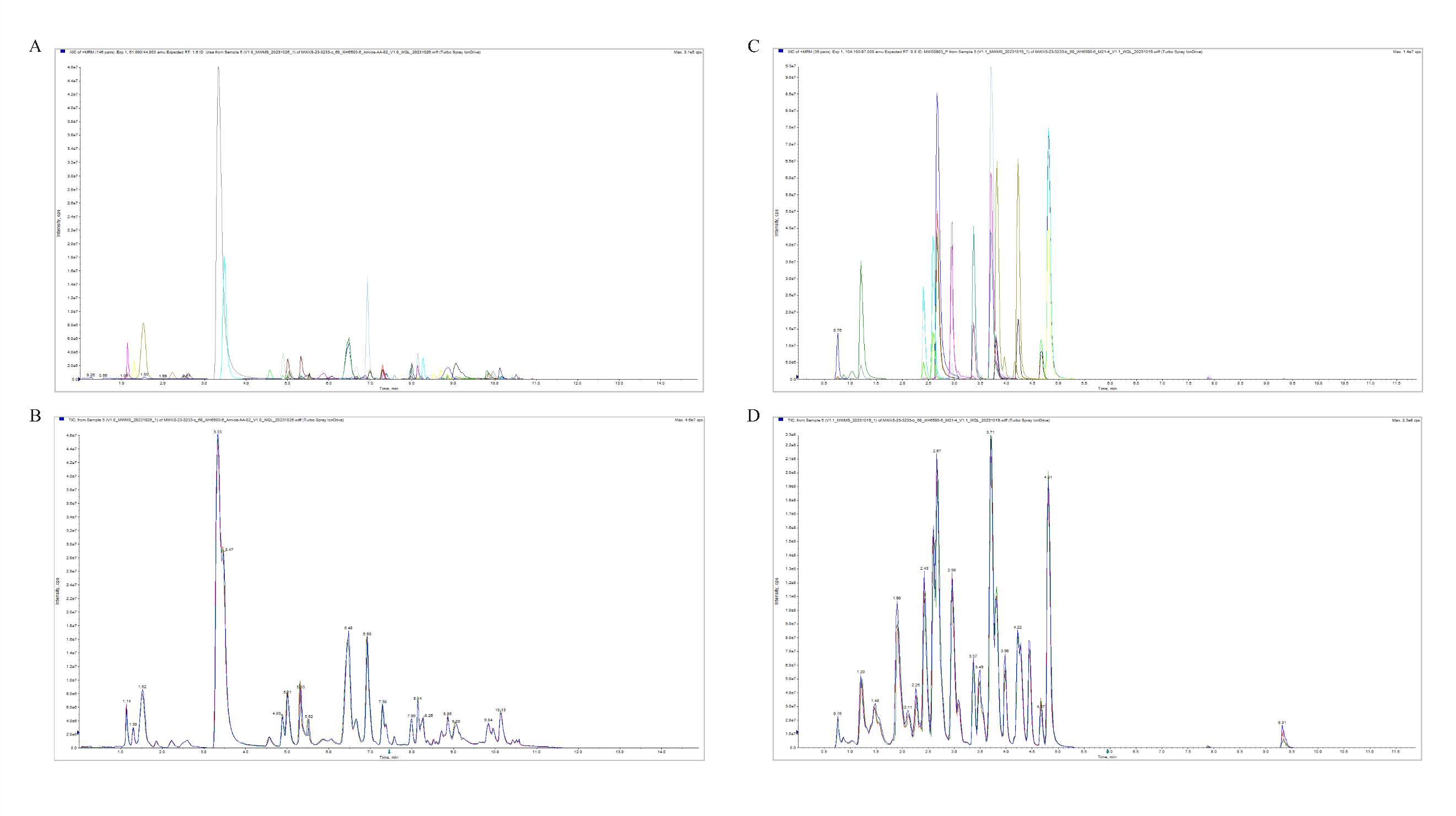


Fig. S3. Free amino acids and derivatives: (A) overlaps of TIC of QC samples MS analysis; (B) Multiplex diagram of MRM metabolite detection (Positive ion mode). Organic acids and derivatives: (C) Overlaps of TIC of QC samples MS analysis; (D) Multiplex diagram of MRM metabolite detection.


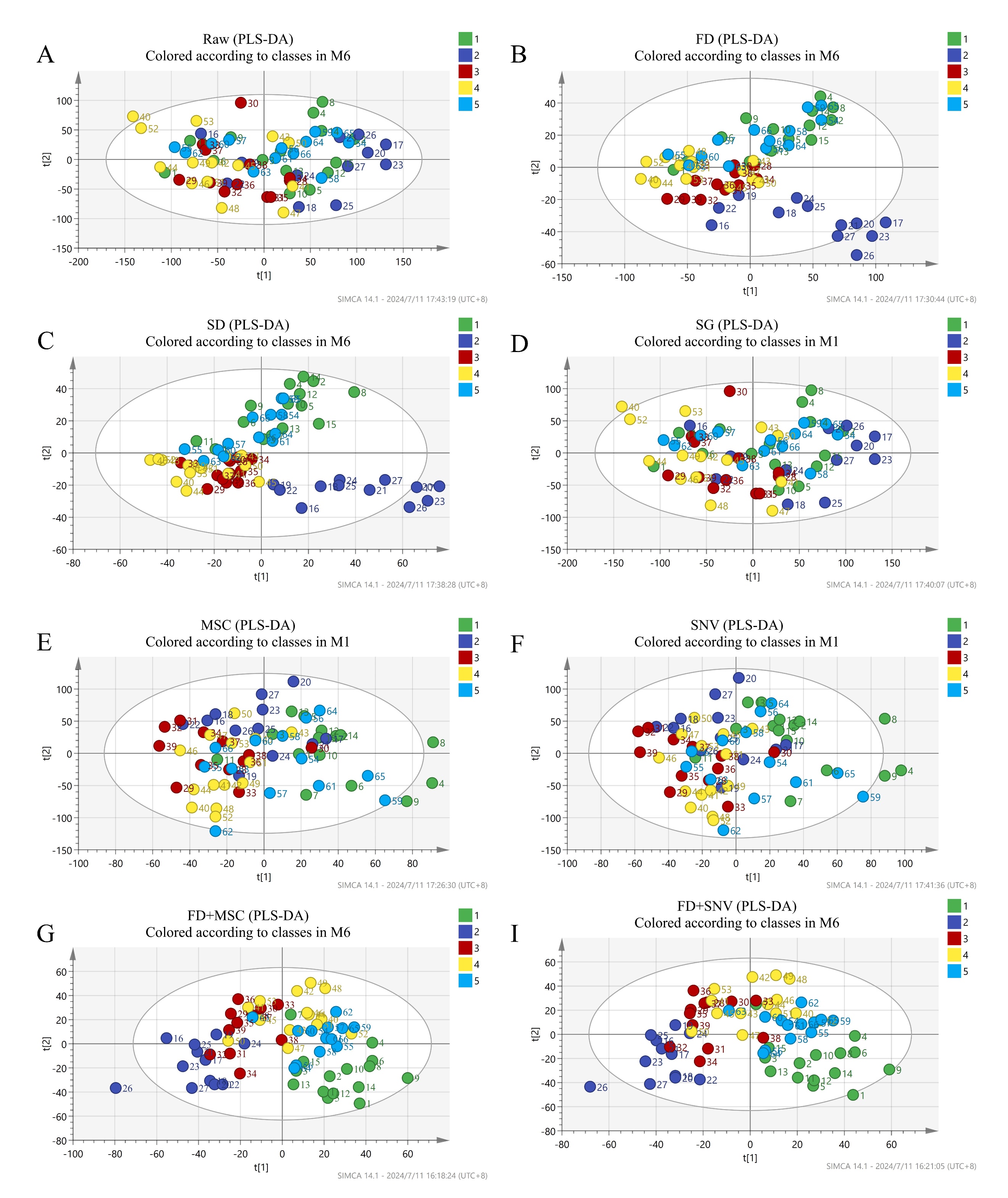


Fig. S4. Scatterplot of PLS-DA model with different preprocessing methods


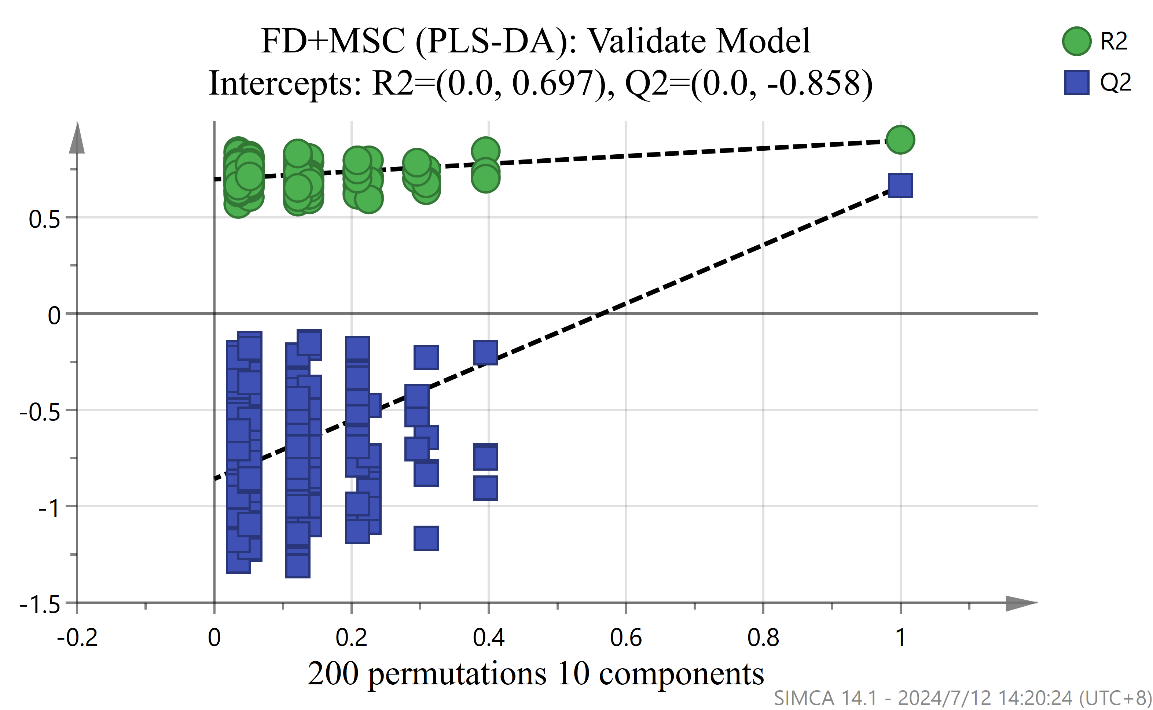


Fig. S5. 200 permutation test results for the best PLS-DA model.
